# Modeling the spread of COVID-19 as a consequence of undocumented immigration toward the reduction of daily hospitalization: Case reports from Thailand

**DOI:** 10.1371/journal.pone.0273558

**Published:** 2022-08-25

**Authors:** Tanatorn Intarapanya, Apichat Suratanee, Sittiporn Pattaradilokrat, Kitiporn Plaimas

**Affiliations:** 1 Advanced Virtual and Intelligence Computing (AVIC) Center, Department of Mathematics and Computer Science, Faculty of Science, Chulalongkorn University, Bangkok, Thailand; 2 Department of Mathematics, Faculty of Applied Science, King Mongkut’s University of Technology North Bangkok, Bangkok, Thailand; 3 Intelligent and Nonlinear Dynamic Innovations Research Center, Science and Technology Research Institute, King Mongkut’s University of Technology North Bangkok, Bangkok, Thailand; 4 Department of Biology, Faculty of Science, Chulalongkorn University, Bangkok, Thailand; Nanyang Technological University, SINGAPORE

## Abstract

At present, a large number of people worldwide have been infected by coronavirus 2019 (COVID-19). When the outbreak of the COVID-19 pandemic begins in a country, its impact is disastrous to both the country and its neighbors. In early 2020, the spread of COVID-19 was associated with global aviation. More recently, COVID-19 infections due to illegal or undocumented immigration have played a significant role in spreading the disease in Southeast Asia countries. Therefore, the spread of COVID-19 of all countries’ border should be curbed. Many countries closed their borders to all nations, causing an unprecedented decline in global travel, especially cross-border travel. This restriction affects social and economic trade-offs. Therefore, immigration policies are essential to control the COVID-19 pandemic. To understand and simulate the spread of the disease under different immigration conditions, we developed a novel mathematical model called the Legal immigration and Undocumented immigration from natural borders for Susceptible-Infected-Hospitalized and Recovered people (*LUSIHR*). The purpose of the model was to simulate the number of infected people under various policies, including uncontrolled, fully controlled, and partially controlled countries. The infection rate was parameterized using the collected data from the Department of Disease Control, Ministry of Public Health, Thailand. We demonstrated that the model possesses nonnegative solutions for favorable initial conditions. The analysis of numerical experiments showed that we could control the virus spread and maintain the number of infected people by increasing the control rate of undocumented immigration across the unprotected natural borders. Next, the obtained parameters were used to visualize the effect of the control rate on immigration at the natural border. Overall, the model was well-suited to explaining and building the simulation. The parameters were used to simulate the trends in the number of people infected from COVID-19.

## 1. Introduction

In December 2019, there were many reports of a novel coronavirus renamed by the World Health Organization (WHO) as COVID-19 on February 11, 2020 [1]. This disease spread quickly, and the number of infected individuals increased rapidly. According to the WHO reports, there were 6,530,992 confirmed cases and 406,665 deaths globally as of June 5, 2020 [2]. One month later, the numbers had doubled to a confirmed 11,172,123 cases and 557,558 deaths globally, as reported on July 5, 2020 [2]. The COVID-19 disease is highly infectious with an average incubation period of 5 to 7 days [3]. Moreover, the virus could quickly spread from asymptomatic patients by direct contact, coughing, sneezing, and talking. The virus could survive in aerosol form or the surfaces or breath droplets for up to 72 hours [4]. Without prompt diagnosis and treatment, COVID-19 could cause severe symptoms and even death [5].

Initially, the first case of COVID-19 was reported in Wuhan, China [6]. Later, due to the global aviation industry and the limited COVID-19 diagnostic tools, the disease spread to several countries. Beginning in January of 2020, tourists from China with COVID-19 were first reported in Thailand [7]. To contain the disease, the Thailand government restricted international air travel, resulting in the containment of the disease and reducing the number of new cases to almost zero. However, since March of 2021, the Thai government reported that the virus was diagnosed on people who had immigrated illegally. Due to inadequate disease prevention measures and poor access to medical diagnostic and treatment, the spread of COVID-19 increased [8]. **Recent studies reported that the high prevalence of COVID-19 and deaths occurred in unauthorized immigrants globally [9, 10]**. As a result, it is necessary to understand and simulate the spread of the disease under different immigration scenarios. The analysis of control rates of illegal immigration should be addressed. Long-term behavior of the situation under a control policy should be studied and revealed to simulate the number of people in different classes of the populations in the country.

To understand the spread of infectious diseases, mathematical models of epidemics were evaluated to simulate the dynamic transmission and plan effective strategies to control the spread. One of the model most often used is the Susceptible-Infected-Recovered (*SIR*) model, which describes the flow of individuals through three subpopulations: susceptible (*S*), infected (*I*), and recovered (*R*) [11–14]. This simple model has been the standard for explaining the spread of disease. Over many years, many subsequent models were developed based on this standard [12, 15, 16]. Recently, a model was developed to study the COVID-19 infection in Italy [17]. The model’s name reflects eight subpopulations: susceptible (*S*) (uninfected), infected (*I*) (asymptomatic infected, undetected), diagnosed (*D*) (asymptomatic infected, detected), ailing (*A*) (symptomatic infected, undetected), recognized (*R*) (symptomatic infected, detected), threatened (*T*) (infected with life-threatening symptoms, detected), healed (*H*) (recovered), and extinct (*E*) (dead) - *SIDARTHE*. The aim of this work is to find the cause of the COVID-19 epidemic and help plan an effective control strategy. The model results also confirm the benefits of mass testing, whenever facilities are available.

In 2020, Din et al. developed a new infectious disease model based on the *SIR* model and some other models [18] and used for explaining the spread of COVID-19 in Thailand. The model’s name reflects six subpopulations: susceptible (*S*), latent (*L*) or a group of infected populations that do not show symptoms, infected populations showing symptoms (*I*), quarantine (*Q*), hospitalized (*H*), and recovered (*R*) - *SLIQHR*. Then, the disease transmission was described by six differential equations. This model worked well with the theoretical data under fixed-parameter constants. Although the model simulated the spread of COVID-19, there were still many factors in the second wave related to people who enter the country without tests or quarantine. The inclusion of Thai people in one population (*S* susceptible) was insufficient to explain and simulate that condition.

At the end of 2020, the second wave of COVID-19 shocked the Thai people again because the number of infected people in the country increased dramatically, especially in Samut Sakhon province, Thailand. There, many illegal immigrants worked at the seafood market [19]. The type COVID-19 carried from Myanmar was identified as the delta strain (or B.1617.2), which is transmitted more quickly and more contagious than the other strains; however, the symptoms were less severe than the original type spreading from Wuhan, China. Currently, the Thailand government controls these conditions by locking down some regions infected with the virus [19]. The control scenarios and the data were recorded as the reference data to model the spread of this disease, expand our knowledge, and understand the situation mathematically. Additionally, the model can help reveal the rate of infections, control the spread from the unprotected natural border, and develop policies and a strategy to protect people from the virus.

Based on the basic concepts of the *SIR* and *SLIQHR* models, we developed a novel compartment diagram to model the spread of COVID-19 under the conditions that some infected people will enter the country through the uncontrolled natural border. The model’s name reflects six subpopulations: Legal immigrants (*L*), Undocumented immigrants from natural border (*U*), Susceptible people (*S*), Infected people (*I*), Hospitalized people (*H*) and Recovered people (*R*) - *LUSIHR*. We also developed another model, *LSIHR*, by setting the value of the control rate of undocumented immigration (*ω*) as 1, removing the natural border population from the *LUSIHR* model. The assessment of the model focused on its positivity, the equilibrium point, and the basic reproduction number (*R*_0_) or the average number of cases generated per infected individual [20] for the *LSIHR* model. The simulation was conducted using various scenarios and control rates. Finally, the simulated results were compared with real collected data in Thailand. More details on the *LUSIHR* and *LSIHR* models are presented in Section 2. Data manipulation and numerical parameters are explained in Section 3. Section 4 describes the results of the well-posedness and mathematical analysis for the equilibrium points, as well as the model simulation results, comparing them to the actual collected data. Section 5 describes the conclusions of this work. Finally, the benefit and limitation of this work are discussed in Section 6.

## 2. The spread of disease model and *LUSIHR* model

### 2.1 Background

The mathematical models of disease spread have been studied for some time. The most basic and simple model is the *SIR* model that describes the virus spread using only three variables. In contrast, currently, much of the derivative family of this base model can be found for a specific condition or disease [12, 15, 16, 21]. Under the conditions in which Thailand faces the COVID-19 disease, the first proposed model *SLIQHR* was reported recently in 2020 [18]. Notice that both models start with a single susceptible group set as the single source of the population in the model. However, based on the conditions of illegal immigration in Thailand, we can add a group of immigrants into the susceptible group. The susceptible group represents all the people who can be expected to be infected in the future. This scenario was proposed in this study as the *LUSIHR* model, described in the following section.

### 2.2 The *LUSIHR* model

**Fig 1:**
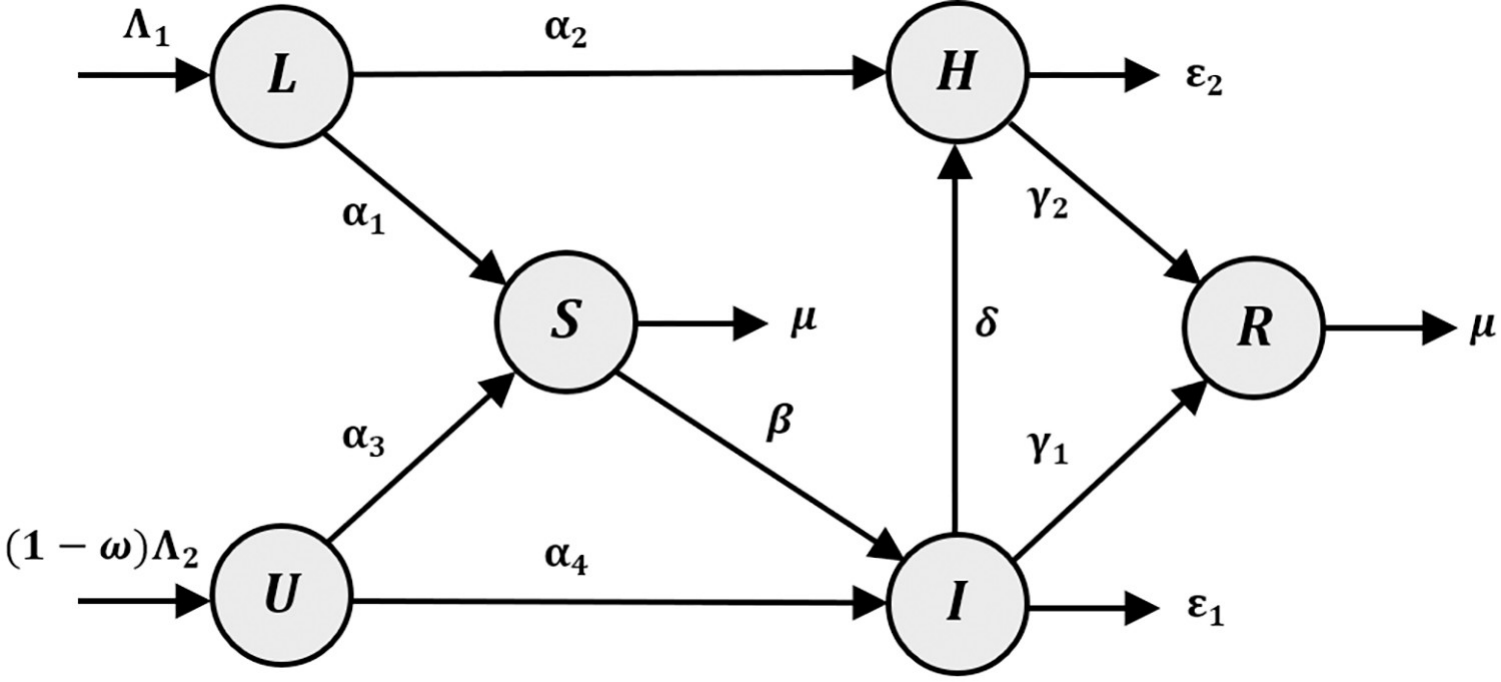
Diagram of the *LUSIHR* model. Nodes represent population classes and edges represent transmissions and their rates.

The *LUSIHR* model is an epidemic model based on the *SIR* and *SLIQHR* models. In principle, the whole population was divided into six subgroups: Legal immigrants (*L*) are people who travel into the country legally, Undocumented or illegal immigrants are people (*U*) who travel across unprotected natural border, Susceptible population (*S*) are those who have not been infected with the disease, Infected populations (*I*) are those who are already infected and not tested for the disease, Hospitalized populations (*H*) are those who are already infected, have tested positive, and are being treated in a hospital, finally, Recovered populations (*R*) refer to those who have recovered from the disease and cannot be infected again. Notice that people who travel into Thailand legally must be tested for the disease and be quarantined until they can travel to all regions of Thailand. Following quarantine, people in this group can move into either susceptible or hospitalized groups. In contrast, people who enter through the natural border enter the country without any test or quarantine. As a result, people in this group can move into either susceptible or infected groups. The diagram of the *LUSIHR* model is shown in Fig 1.

The diagram of the *LUSIHR* model (in Fig 1) represents the states of the people in Thailand. At a specific time ***t***, the number of populations in each group could be changed over time. Let *T*(*t*) be the total population at time *t*. For any *t*≥0, we then have *T*(*t*) = *L*(*t*)+*U*(*t*)+*S*(*t*)+*I*(*t*)+*H*(*t*)+*R*(*t*) where *L*(*t*), *U*(*t*), *S*(*t*), *I*(*t*), *H*(*t*), and *R*(*t*) are the number of legal immigrants, undocumented immigrants from the natural border, susceptible people, infected people, hospitalized people, and recovered people over time *t*. Based on the diagram, the system of ordinary differential equations for the *LUSIHR* model can be written as follows:

$$L^{\prime }(t)=\Lambda_{1}-\alpha_{1}L(t)-\alpha_{2}L(t)$$ (1)

$$U^{\prime }(t)=\Lambda_{2}(1-\omega )-\alpha_{3}U(t)-\alpha_{4}U(t)$$ (2)

$$S^{\prime }(t)=\alpha_{1}L(t)+\alpha_{3}U(t)-\beta S(t)I(t)-\mu S(t)$$ (3)

$$I^{\prime }(t)=\alpha_{4}U(t)+\beta S(t)I(t)-\delta I(t)-\varepsilon_{1}I(t)-\gamma_{1}I(t)-\mu I(t)$$ (4)

$$H^{\prime }(t)=\alpha_{2}L(t)+\delta I(t)-\varepsilon_{2}H(t)-\gamma_{2}H(t)$$ (5)

$$R^{\prime }(t)=\gamma_{1}I(t)+\gamma_{2}H(t)-\mu R(t)$$ (6)

The model parameters are as follows:

Λ_1_ is the number of recruitments per day into legal immigration group (*L*).

Λ_2_ is the number of recruitments per day into undocumented immigration from the natural border (*U*).

*ω* is the control rate of undocumented immigration from the natural border.

*α*_1_ is the proportion of people transfers from legal immigration group (*L*) to susceptible group (*S*).

*α*_2_ is the proportion of people transfers from legal immigration group (*L*) to hospitalized group (*H*).

*α*_3_ is the proportion of people transfers from the undocumented immigrant group (*U*) to the susceptible group (*S*).

*α*_4_ is the proportion of people transfers from the undocumented immigrant group (*U*) to the infected group (*I*).

*γ*_1_ is the recovery rate per unit of time of the infected people.

*γ*_2_ is the recovery rate per unit of time of the hospitalized people.

*β* is the transmission rate per unit of time of the susceptible people who interact with infected people.

*δ* is the proportion of infected group (*I*) aware that they are infected and tested at hospital.

*ε*_1_ is the death rate per unit of time of the infected people.

*ε*_2_ is the death rate per unit of time of the hospitalized people.

*μ* is the rate per unit of time of the people traveling out of the country.

**Fig 2:**
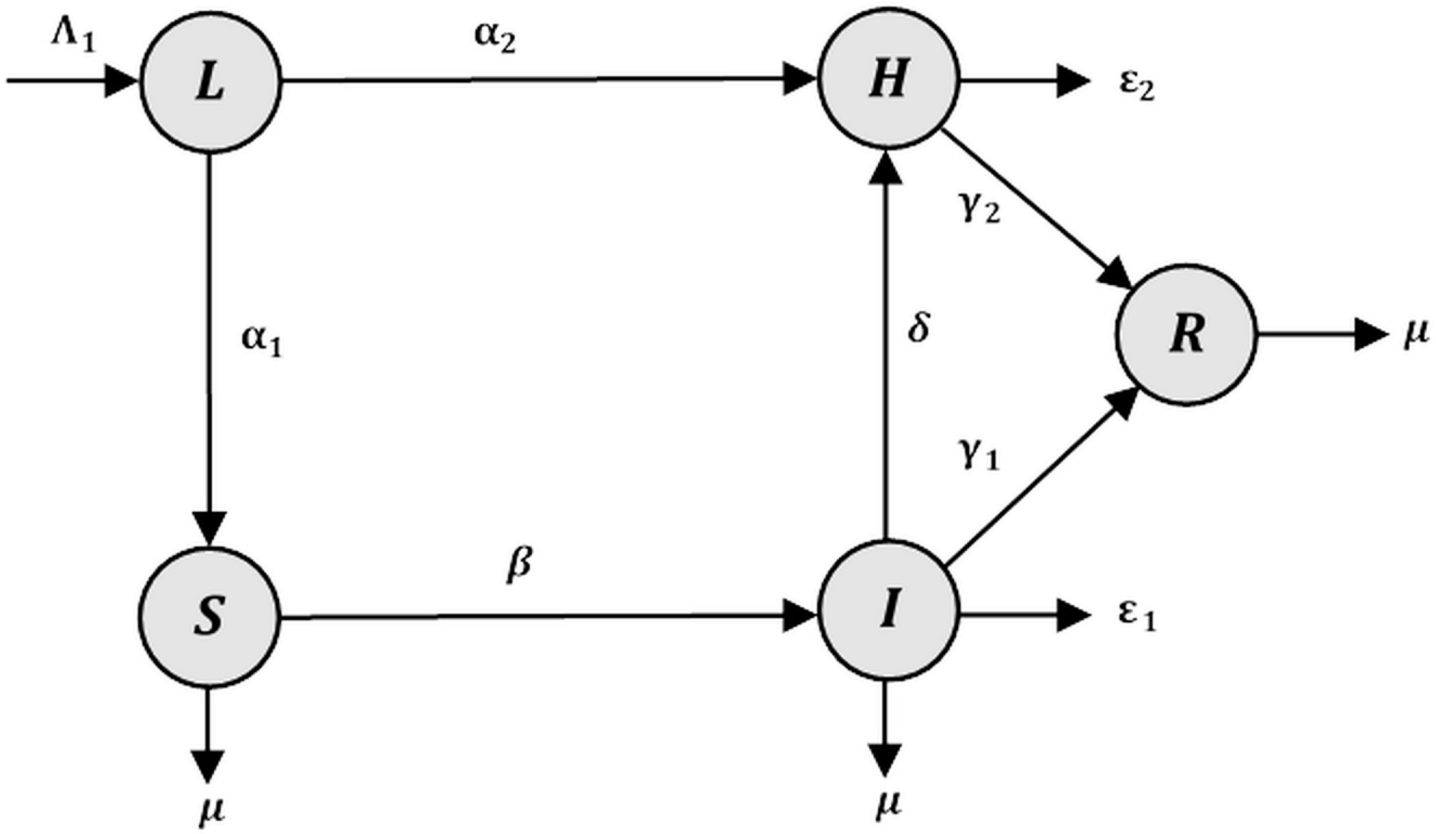
Diagram of the *LSIHR* (Legal immigration–Susceptible–Infected–Hospitalized—Recovered) model. Nodes represent population classes and edges represent transmissions and their rates.

Notice that the control rate of undocumented immigration at natural border (*ω*) was introduced to measure the success of efforts to slow the rate of immigration at Thailand’s borders. This rate was assigned a value from 0 to 1. In those cases where there is no control at the border, *ω* was set to zero. On the other hand, if *ω* = 1, no one could enter the country via the natural border. As a result, the natural border subpopulation was removed from the *LUSIHR* model and placed in the *LSIHR* model. Therefore, the control rate was divided into two parts, 0≤*ω*<1 for the *LUSIHR* model, and *ω* = 1 for the *LSIHR* model. The diagram of the *LSIHR* model is shown in Fig 2.

The system of ordinary differential equations for the *LSIHR* model is different from the *LUSIHR* model by removing the undocumented immigrants from the natural border and introducing new equations for the susceptible people and infected people. Therefore, the system of the differential equations for the *LSIHR* model derived from the *LUSIHR* by replacing Eqs (3) and (4) with the following Eqs (7) and (8):

$$S^{\prime }(t)=\alpha_{1}L(t)-\beta S(t)I(t)-\mu S(t)$$ (7)

$$\mathrm{and}\;I^{\prime }(t)=\beta S(t)I(t)-\delta I(t)-\varepsilon_{1}I(t)-\gamma_{1}I(t)-\mu I(t).$$ (8)

## 3. Data manipulation and numerical parameters

### 3.1 Data sources

The Department of Disease Control, Ministry of Public Health, Thailand, provided the data used in this study [19]. The data includes the number of people in quarantine, infected people, recovered people, hospitalized people, infected migrant workers, and deaths. We focused on the data for hospitalized people from December 1, 2020 (Day 1) to February 23, 2021 (Day 85) (see S1 Table). The data for the number of people who immigrated via the natural border route and via legal immigration was obtained from the Immigration Bureau [22].

### 3.2 Numerical solution

To parameterize the *LUSIHR* model, it is necessary to identify the optimal parameters to use in the model equations. Some parameters, including Λ_1_,Λ_2_,*ω*,*α*_1_,*α*_2_,*α*_3_,*α*_4_,*γ*_1_,*γ*_2_,*ε*_1_,*ε*_2_, and *μ*, can be estimated using the actual data. The estimated value of each parameter is shown in Table 1.

**Table 1:** The value of each parameter in the model.

Parameters	Value
Λ_1_	5219.77 persons/days
Λ_2_	297.5 persons/days
*ω*	0.4
*α*_1_	0.9971
*α*_2_	0.0029
*α*_3_	0.7484
*α*_4_	0.2516
*δ*	0.3
*μ*	0.00230154

The number of people who illegally immigrated daily (Λ_1_) and the rate of the people traveling out of the country (*μ*) were calculated based on the average number of tourists recorded in an Immigration Bureau report of December 2020 [22]. We assumed that the control rate of undocumented immigration over the natural border (*ω*) was approximately 0.4. Actually, it is impossible to approximate the actual value of *ω* because only the number of arrested immigrants can be recorded. Obviously, the total number of undocumented or illegal immigrants should be considered but it is hard to know this number. Therefore, let’s assume that about half or lower a half would be an approximated rate that cause an effect to the situation. It means that the number of illegal immigrants arrested at the natural border was 40% of the expected total illegal immigrants. From this, we calculated the number of people who immigrated via natural border daily (Λ_2_). The value of *α*_2_ was calculated using the ratio of the total imported cases to the total number of people entering the country in December 2020. Similarly, *α*_4_ was calculated using the ratio of the total infected migrant workers to the expected total people entering the country illegally in December 2020. The values of *α*_1_ and *α*_3_ were 1−*α*_2_ and 1−*α*_4_, respectively. The value *δ* was assumed to be 0.3.

Because the values of *γ*_2_ and *ε*_2_ were for hospitalized people, they were calculated using the data from each region. The remaining parameters were difficult to obtain because they are relevant to the infectious disease represented by infected people; however, no tests or data from the hospitals were available. Therefore, the optimal parameters were obtained by minimizing the error between the actual data and simulated data. The loss function that we used for the error was the root mean square error (*RMSE*). The value of *RMSE* was always non-negative, and closer to zero is better. The formula for the RMSE is:

$$RMSE=\sqrt{\frac{\sum_{i=1}^{N}{\left(x_{i}-{\hat{x}}_{i}\right)}^{2}}{N}}$$

where

*RMSE* is the value of the root mean square error,

*N* is the number of non-missing data points, in this model, the total number of days,

*x*_*i*_ is actual data at day *i*, and
${\hat{x}}_{i}$ is simulated data at day *i*.

## 4. Results

### 4.1 The nature of model parameters

For the *LUSIHR* and *LSIHR* models, since the number of each subpopulation could not be negative, it was essential to prove that the solutions were non-negative (all populations more than or equal to zero) if the initial conditions were positive. The model positivity for these two model can be derived from the proof of the model positivity for the feedback vaccination law for *SIR* model [21]. The population functions *L*(*t*), *U*(*t*), *S*(*t*), *I*(*t*), *H*(*t*), and *R*(*t*) are continuous and differentiable as shown in Eqs (1)—(8). Then, if the population decreased to zero, the derivative was positive to increase the population to be positive again. With this concept, the proof of these two models was shown in S1 Appendix (Appendix A2: the proof of model positivity). Therefore, all the solutions are non-negative for all times and these two proposed models can be applied to analyze its equilibrium points further and simulate the growth of the populations.

### 4.2 The equilibrium points

A system has an equilibrium point if there is no change in the system at all times. Commonly, there are two types of equilibrium points: an endemic equilibrium point (EE) and a disease-free equilibrium point (DFE). The EE point is the state that the spread of disease still exists all the time. On the other hand, the DFE point is the state at which the infection disappears from the system at all times.

For the *LUSIHR* model, the equilibrium point was calculated by setting Eqs (1)—(6) equal to zero as a non-linear system. The way to find the solution is represented in S1 Appendix (Appendix A3: The analysis of an equilibrium points). We obtained only one EE point in which the infection was not removed from the system as

$$\left(L,U,S,I,H,R)=(L^{*},U^{*},S^{*},I^{*},H^{*},R^{*}\right)$$

where $L^{*}=\frac{\Lambda_{1}}{\alpha_{1}+\alpha_{2}}$, $U^{*}=\frac{\Lambda_{2}(1-\omega )}{\alpha_{3}+\alpha_{4}}$, $S^{*}=\frac{C-\sqrt{C^{2}-4\mu \beta AB}}{2\mu \beta }$, $I^{*}=\frac{\alpha_{4}U^{*}}{A-\beta S^{*}}$, $H^{*}=\frac{\alpha_{2}L^{*}+\delta I^{*}}{\varepsilon_{2}+\gamma_{2}}$, and $R^{*}=\frac{\gamma_{1}I^{*}+\gamma_{2}H^{*}}{\mu }$, with *A* = *δ*+*ε*_1_+*γ*_1_+*μ*, *B* = *α*_1_L*+*α*_3_U* and *C* = *μA*+*βB*+*α*_4_βU*.

For the *LSIHR* model, we obtained two equilibrium points (see S1 Appendix (Appendix A3: The analysis of an equilibrium points)), one of which was the EE point and the other was the DFE point. The DFE point of the *LSIHR* model is

$$\left(L,S,I,H,R)=(L_{1}^{*},S_{1}^{*},I_{1}^{*},H_{1}^{*},R_{1}^{*}\right)$$

where $L_{1}^{*}=\frac{\Lambda_{1}}{\alpha_{1}+\alpha_{2}}$, $S_{1}^{*}=\frac{\alpha_{1}L^{*}}{\mu }$, $I_{1}^{*}=0$, $H_{1}^{*}=\frac{\alpha_{2}L^{*}+\delta I^{*}}{\varepsilon_{2}+\gamma_{2}}$, and $R_{1}^{*}=\frac{\gamma_{1}I^{*}+\gamma_{2}H^{*}}{\mu }$. Note that the DFE point of the *LSIHR* should have I* = 0 in all components of the DFE.

The EE point of the *LSIHR* model is

$$\left(L,S,I,H,R)=(L_{2}^{*},S_{2}^{*},I_{2}^{*},H_{2}^{*},R_{2}^{*}\right)$$

where $L_{2}^{*}=\frac{\Lambda_{1}}{\alpha_{1}+\alpha_{2}}$, $S_{2}^{*}=\frac{A}{\beta }$, $I_{2}^{*}=\frac{\alpha_{1}L^{*}}{A}-\frac{\mu }{\beta }$, $H_{2}^{*}=\frac{\alpha_{2}L^{*}+\delta I^{*}}{\varepsilon_{2}+\gamma_{2}}$, and $R_{2}^{*}=\frac{\gamma_{1}I^{*}+\gamma_{2}H^{*}}{\mu }$, with *A* = *δ*+*ε*_1_+*γ*_1_+*μ*.

Because the *LUSIHR* model has only one EE point, the infected group did not disappear from the system. The best option was to reduce the number of infected people as much as possible. One way to reduce the number was by increasing the value of the control rate of undocumented immigration over the natural border (*ω*). On the other hand, the *LSIHR* model was formed by setting *ω* = 1, the maximum value of the control rate. This model had two equilibrium points, the DFE point, and the EE points. Under actual conditions, we sought to reduce the infection and converge to the DFE point so that the infection disappeared from the system. The condition that the population trajectory would converge to DFE or EE depended on the basic reproduction number (*R*_0_).

### 4.3 The local stability of the equilibrium points and the basic reproduction number (*R*_0_)

The stability of the DFE and the EE points was determined by the condition of eigenvalues [23, 24]. To obtain the eigenvalue, the Jacobian was built and considered at each equilibrium point (see S1 Appendix (Appendix A4)). The results in Appendix A4 in S1 Appendix show us that both systems eventually converge to the equilibrium points. To classify the type of the equilibrium points, the basic reproduction number (*R*_0_) helps us to better understand the stability of the system. *R*_0_ is the basic reproduction number, meaning the average number of cases generated per infected individual [20]. When *R*_0_>1, infections will spread in a trajectory that converges at the EE point. However, when *R*_0_<1, infections will decrease over time and the number of infected people will reach the DFE point. For the *LUSIHR*, the system is always stable and towards to the EE point while the *LSIHR* model provides two phase transitions: the EE point and DFE point as shown in S1 Appendix (Appendix A4).

### 4.4 Simulation results

This part presents an example based on specific parameters to simulate the population growth with the *LUSIHR* and *LSIHR* models. The results are compared to the values for the simulation trajectory trend to the EE point for the *LUSIHR* model calculated previously. For the *LSIHR* model, we gave examples for the DFE and EE depending on the value of the basic reproduction number (*R*_0_). These simulations were conducted using the MATLAB computing platform.

Assume that the initial values of all variables for *LUSIHR* model are as follows:

L(0)=0,U(0)=0,S(0)=15000,I(0)=20,H(0)=0,andR(0)=0.

The values of all parameters are set as Λ_1_ = 30, Λ_2_ = 15, *ω* = 0, *α*_1_ = 0.7, *α*_2_ = 0.02, *α*_3_ = 0.3, *α*_4_ = 0.2, $\beta =\frac{0.2}{15000},$ *δ* = 0.1, *γ*_1_ = 0.002, *γ*_2_ = 0.005, *ε*_1_ = 0.01, *ε*_2_ = 0.008 and *μ* = 0.001.

The simulation began with only the susceptible and infected populations. Initially, the number of susceptible persons was set at 15,000 people, and the number of infected persons was 20. Approximately 30 people per day were assumed to enter the country legally, and 15 people per day entered the country illegally without testing or quarantine. The control rate of undocumented immigration across the natural border was set at zero, meaning there were no controls at the border. About 70% of legal immigrants were assumed to be susceptible to the virus (*α*_1_ = 0.7) and 2% were hospitalized (*α*_2_ = 0.02). The percentage of people crossing the natural border who were susceptible and infected was 30% and 20% (*α*_3_ = 0.3, *α*_4_ = 0.2), respectively. The transmission rate of the infection was 0.2/15000 people daily who were susceptible after interacting with infected people. Approximately 10% of infected people were assumed to be hospitalized. The daily recovery rate for infected and hospitalized people was 0.002 and 0.005, respectively. The daily death rate for infected and hospitalized people was 0.01 and 0.008, respectively. Finally, the daily rate for susceptible, infected, and recovered populations emigrating from the country was 0.001.

**Fig 3:**
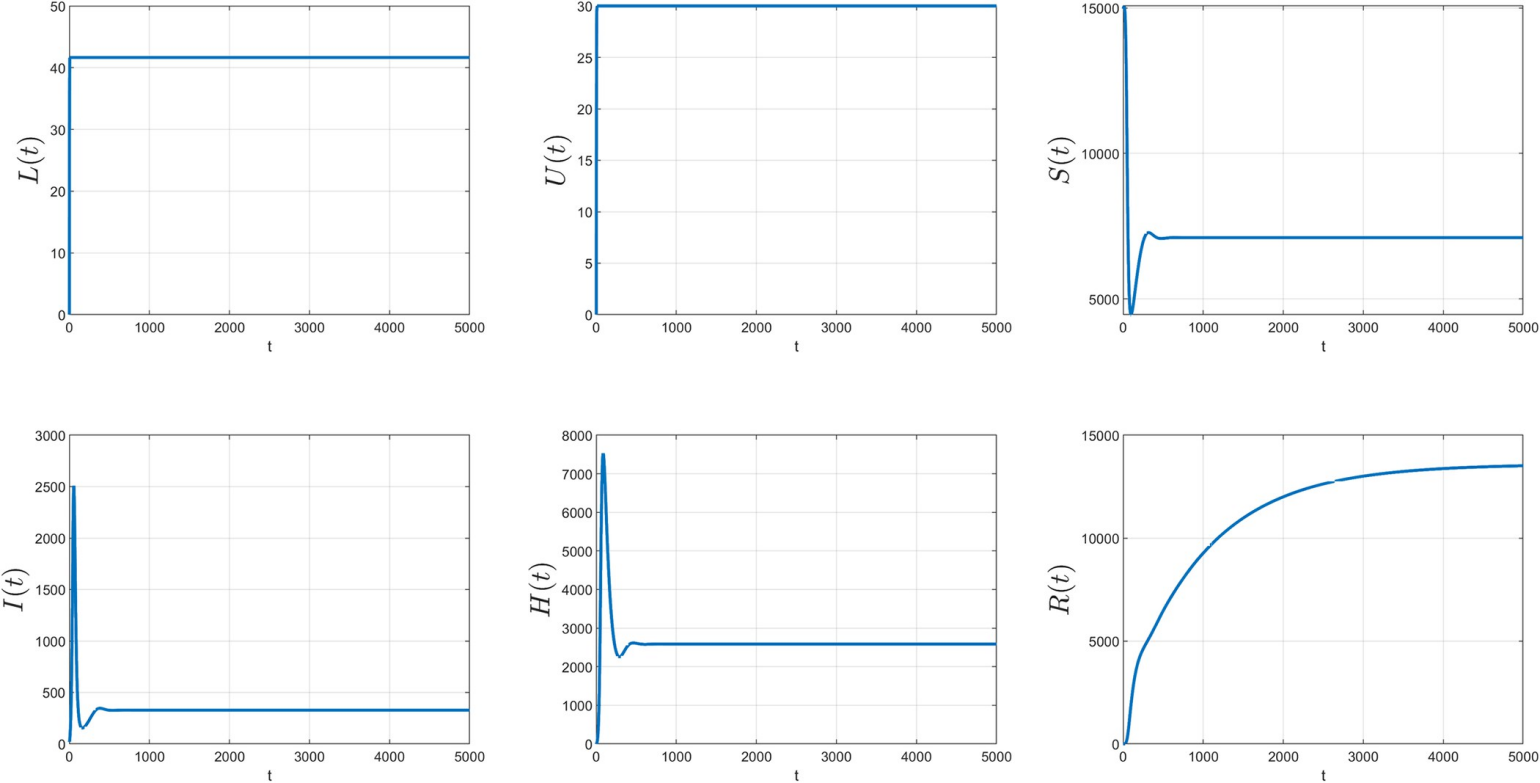
The simulation results of the LUSIHR model. Each plot represents each subpopulation on the y axis and time in days on the x axis.

In the long run, the simulation resulted in the same equilibrium points calculated in the analysis, with the same parameters and initial values. The plot of the simulation results is shown in Fig 3. The EE point (L*, U*, S*, I*, H*, R*) = (41.667, 30, 7103, 327.9967, 2523.9, 12849) meant that in the long run, stability was achieved at 41.667 for the legal border, 30 for the natural border, 7,103 for susceptible persons, 327.9967 for infected persons, 2,523.9 for hospitalized persons, and 12,849 for recovered populations.

As shown in Fig 3, the solution trajectory trends to the EE point calculated previously. The simulation results confirmed that the analyzed equilibrium point was accurate in explaining the conditions. We could use this equilibrium point to calculate the state of the population in the long run if we have a non-negative initial condition of the population and specific values for the parameters. For the simulations using the *LSIHR* model, the initial values were

L(0)=0,S(0)=15000,I(0)=20,H(0)=0,andR(0)=0.

**Fig 4:**
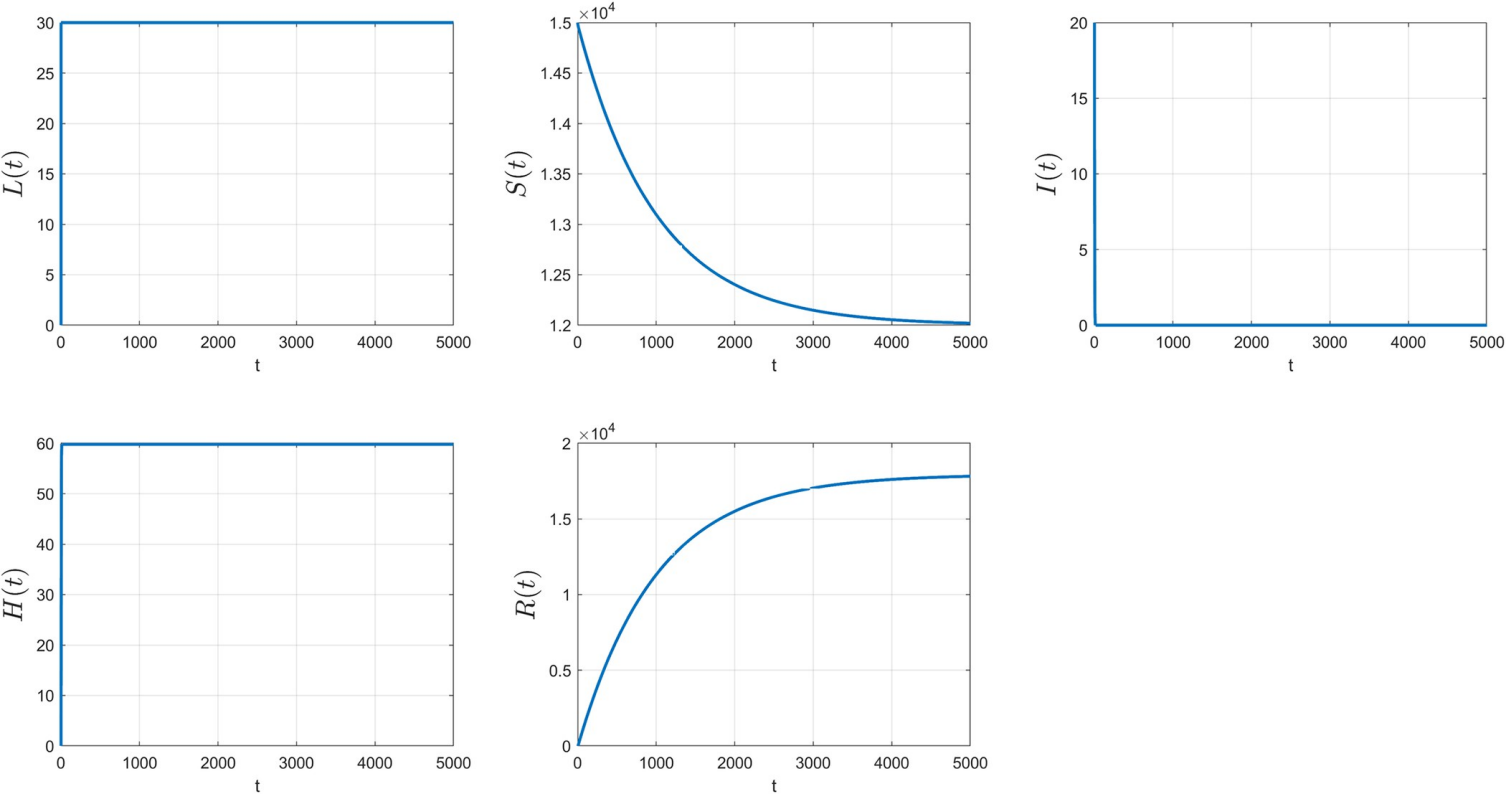
The DFE simulation results of the LSIHR model. Each plot represents each subpopulation on the y axis and time in days on the x axis.

The parametric values for the DFE were Λ_1_ =30, *α*_1_ = 0.4, *α*_2_ = 0.6, $\beta =\frac{0.2}{15000},$ *δ* = 0.6, *γ*_1_ = 0.01, *γ*_2_ = 0.3, *ε*_1_ = 0.03, *ε*_2_ = 0.001 and *μ* = 0.001, which *R*_0_ = 0.2576<1. The simulation results are shown in Fig 4. The DFE point was $\left(L_{1}^{*},S_{1}^{*},I_{1}^{*},H_{1}^{*},R_{1}^{*}\right)$ = (30, 12000, 0, 59.801, 17940).

**Fig 5:**
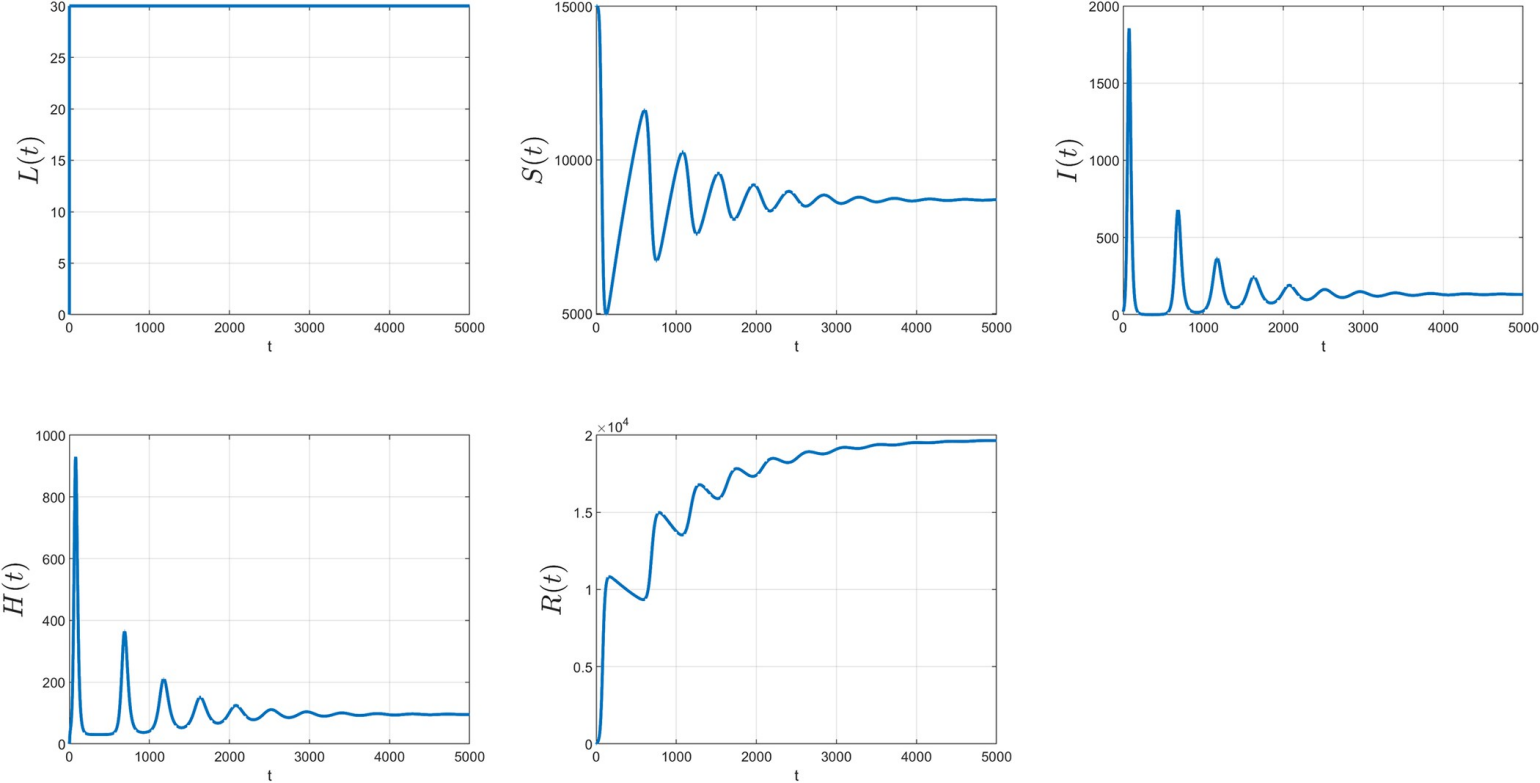
The EE simulation results of the LSIHR model. Each plot represents each subpopulation on the y axis and time in days on the x axis.

The parametric values for the EE were Λ_1_ = 30, *α*_1_ = 0.8, *α*_2_ = 0.2, $\beta =\frac{0.2}{15000},$ *δ* = 0.1, *γ*_1_ = 0.005, *γ*_2_ = 0.2, *ε*_1_ = 0.01, *ε*_2_ = 0.001 and *μ* = 0.001, which *R*_0_ = 2.7586>1 The simulation results are shown in Fig 5. The EE point was $\left(L_{2}^{*},S_{2}^{*},I_{2}^{*},H_{2}^{*},R_{2}^{*}\right)$ = (30, 8700, 131.9, 95.471, 19754).

The *LSIHR* model, which represents the *LUSIHR* model with ***ω*** = **1**, is a model for no undocumented immigrants entering into the country. Notice that when all parameters are constants but only the proportion of people transfers from legal immigration (*α*_1_) and the transmission rate per unit of time of susceptible people interacting with infected people (*β*) vary. The spread of the COVID-19 still occurs in the case that *α*_1_ is large enough to make ***R***_**0**_>**1**. It means that the system contains more susceptible cases which lead to more contact and interactions with the infected people and cause the pandemic. In the same manner, if *β* is large meaning that it is a fast and high transmission rate when people contact to an infected people, the pandemic will occur. Obviously, ***R***_**0**_>**1** as shown in Fig 5. On the other hand, if these two parameters are smaller enough, then ***R***_**0**_<**1** (in Fig 4) which causes a disease-free equilibrium (DFE) eventually and the spread of the COVID-19 can be controlled. On the other hand, in *LUSIHR* model, the analysis shows that only endemic equilibrium (EE) occurs over time. However, there are many factors to curb the spread of COVID-19. Each parameter in the model can help to see the control of the spread of the disease if, in practice, a policy measure would be released to yield the parameter values fitting to the disease-free equilibrium.

### 4.5 Subdivided time intervals for calculating parameters relative to specific conditions

**Fig 6:**
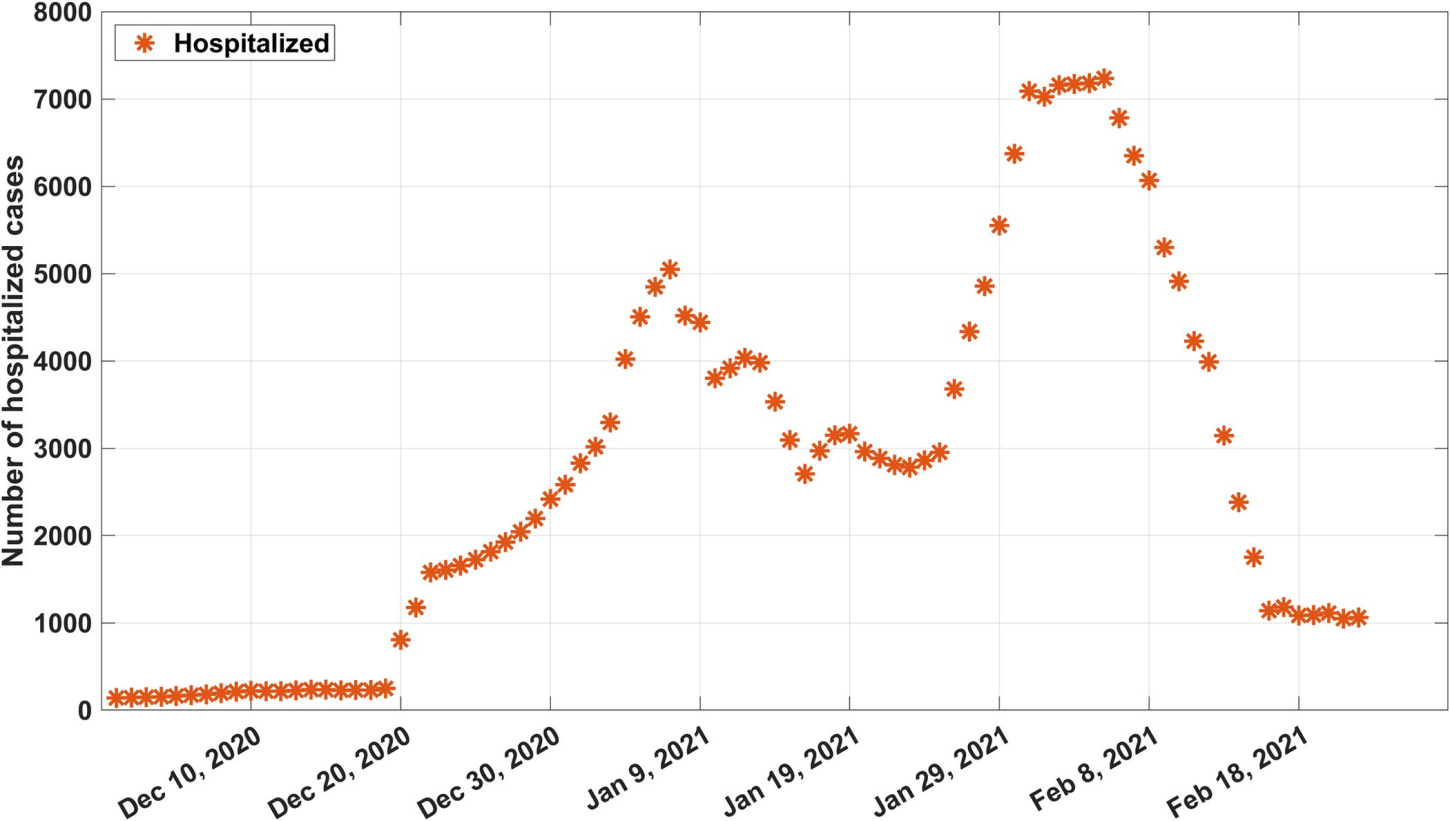
Number of hospitalized people per day. The star dots represent the number of hospitalized cases reported daily.

Based on the recorded data, the number of hospitalized people was of interest. The plot of the growth of these numbers is shown in Fig *6*. Of particular interest were the effect of different conditions, changing behaviors of the people, and the rate of COVID-19 infections in Thailand since the government had issued various containment measures to control the spread. Therefore, the growth period could be divided into four subintervals:

- Day 1–38: the curve continued to increase due to more hospitalized people entering the country illegally across the natural border.
- Day 39–53: the number of hospitalized people decreased due to the entire country being under a locked-down state.
- Day 54–64: the number of hospitalized people increased rapidly due to a domestic super spreader event in a night club caused by non-compliance with the strict lockdown measure.
- Day 65–85: the final interval shows that the number of hospitalized people decreased after Samut Sakhon announced the closure of the shrimp market to prevent the spread of COVID-19.

Table 2 shows the government policy and containment measures issued during these four subintervals. The epidemic had started when some undocumented immigrants entering Thailand approximately at the beginning of December 2020. Consequently, some infected immigrants who worked at a seafood market were the spreaders causing the epidemic heavily in Samut Sakhon province. The government then raised the level of strict preventive measures by dividing area of the country for different levels of control during Interval I. Then, Interval II, the number of hospital people were reduced because of the locked-down state and people were requested to avoid or refrain from travelling across the provinces. Only the person who passes the screening could be travelled. Then, during the joint between Interval II and III, the whole country began to relax but still under the strict control. People were asked to apply the “D-M-H-T-T” measures. However, some people against the rules and were super spreaders causing the second wave of the epidemic. Therefore, the early time in Interval IV, the government then started specific strategies with participatory approaches (called “Bubble and Seal principle”) to control the center of the infection. Thus, the number of hospitalized people were decreasing in this interval.

**Table 2:** Containment measures issued by the government during the four subintervals.

Interval	Containment measures
Interval I: 1st (Dec. 01, 2020)– 38th day	• The Center for COVID-19 Situation Administration (CCSA) had raised the level of strict preventive measures against COVID-19 by dividing area of the country into 4 types: 1) Maximum Control Area, 2) Control Area, 3) High Level Surveillance Area, and 4) Surveillance Area.
Interval II: 39th– 53rd day	• The CCSA requested the collaboration from all people to avoid or refrain from travelling across the provinces except the person who passes the screening.
Interval III: 54th– 64th day	• The establishments and public service centers in Bangkok and other provinces had begun to relax. Still, the measures of the Ministry of Public Health are strictly controlled. • People should apply Department of Health’s measures which is “D-M-H-T-T” as follows; M: wearing a cloth Mask or face Mask all the time, H: wash your Hands frequently with water and soap or alcohol gel, T: Testing for COVID-19 and measuring body Temperature, and the last T: scanning Thai Cha Na application before entering in–out of public places every time and clean body immediately when returning home.
Interval IV: 65th– 85th day (Feb. 23, 2021)	• The Ministry of Public Health had designed a specific strategy; especially, for an infected province, Samut-Sakhon, by using COVID-19 management in the factory with participatory approaches (called “Bubble and Seal principle”) to control the infection not to spread outside.

* Data from the website of Department of Disease Control, Ministry of Public Health, Thailand (https://ddc.moph.go.th/viralpneumonia/eng/situation_more.php).

**Fig 7:**
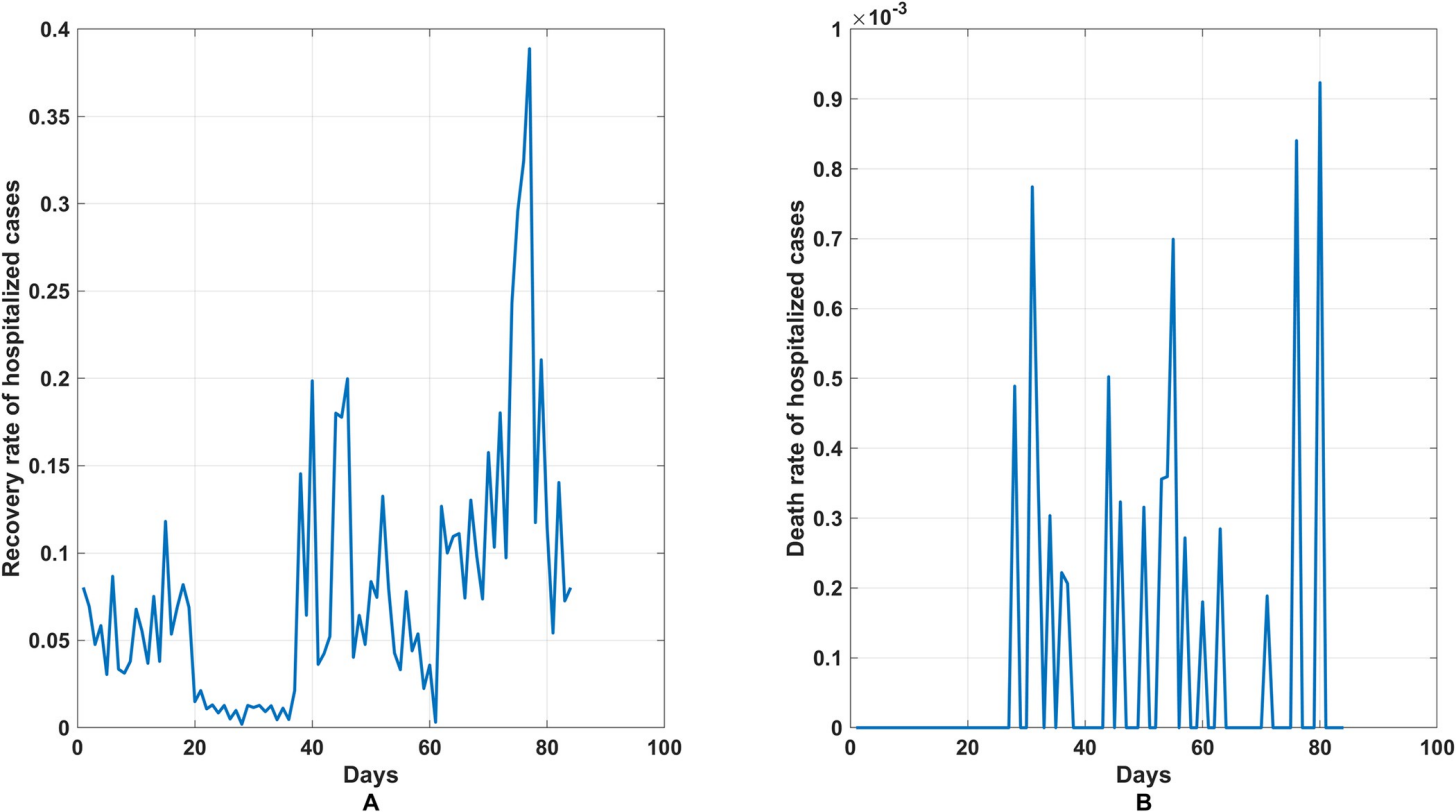
The daily recovery rate and the daily induced death rate for hospitalized people. (A) The daily recovery rate of hospitalized people. (B) The daily induced death rate of hospitalized people.

**Fig 8:**
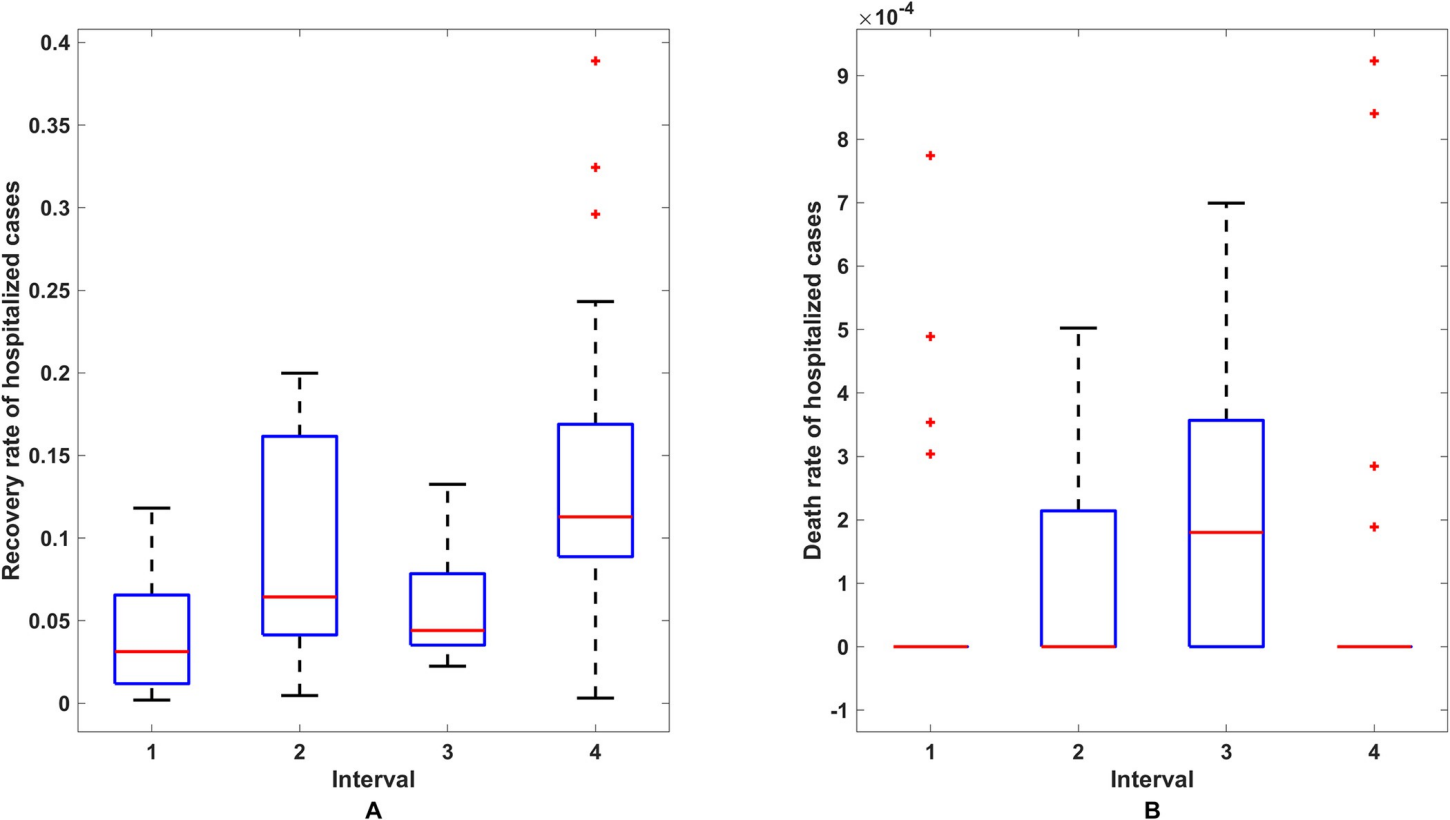
The box plot of the recovery rate and induced death rate for hospitalized people in each interval. (A) The box plot of the recovery rate of hospitalized people for each interval. (B) The box plot of the induced death rate of hospitalized people for each interval.

Notice that the recovery rate of hospitalized people per day (*γ*_2_) could be calculated from the ratio of the newly recovered people to the current hospitalized people. In the same manner, the death rate of hospitalized people per day (*ε*_2_) could be calculated from the ratio of new deaths to currently infected people. The trends of these two rates in each time step (in days) are shown in Fig 7. To examine the rate for each interval, the average value for a specific time interval was calculated and applied in the model. Fig 8 shows the box plot of the values for these two rates for each time interval.

As shown in Fig 8, the median recovery rate and death rate in the first interval were 0.03 and 0, 0.064 and 0 in the second interval, 0.043 and 0.00009 in the third interval, and 0.113 and 0 in the last interval.

The values for the infection rate (*β*), the recovery rate of infected people (*γ*_1_) and the death rate of infected people (*ε*_1_) were then obtained by performing a grid search to minimize the RMSE between the actual data and the model with the median value of the recovery rate of hospitalized people (*γ*_2_) and the induced death rate of hospitalized people (*ε*_2_) for each interval (see Section 3.2 Numerical solution for more detail). The results are shown in Table 3.

**Table 3:** The values of the parameters fitted to the model.

Time Parameter	Interval 1	Interval 2	Interval 3	Interval 4
**Infection rate (*β*),**	2.21×10^−6^	9×10^−9^	2.59×10^−6^	3.73×10^−8^
**Recovery rate of infected people (*γ*_1_)**	4.16×10^−5^	9.34×10^−3^	2.18×10^−4^	3.8×10^−2^
**Death rate of infected people (*ε*_1_)**	2.33×10^−5^	4.59×10^−3^	1.05×10^−4^	1.91×10^−2^

For the behavior of the infection, we expected the values of parameter to be the same in its partition and different for other partitions. Because we divided the time into four intervals depending on the behavior of the infection, we sought to obtain the optimal representative values for the parameters of each interval in the model, as shown in Figs 7 and 8. Under real conditions, it was easy to use the most detailed parameters possible to explain the behavior of the spread. Therefore, the time was divided into four intervals.

In Table 3, for Interval 3, the rate of the COVID-19 transmission (*β*) was increased since there were higher interactions to the infected people due to the night club event. When *β* is large enough, eventually the model turns to an endemic equilibrium. With all four intervals of time, the model reflects the cause of the spread based on illegal immigrations. The second wave in Interval 3 occurred because of not only the illegal immigrations but also the domestic event that some people were non-compliance with the rules. These two coincident events (domestic event and illegal immigration) cause the spread of the COVID-19 in Interval 3. In fact, the cause of the nation people was infected with COVID-19 at that time originally from the undocumented immigrants entering from natural borders. However, it would be a valuable study if these two causes were then separately modelled. The cause of a night club might be modelled by the *LSIHR* model with more reference data supported.

### 4.6 The *LUSIHR* model compared to the actual data

**Fig 9:**
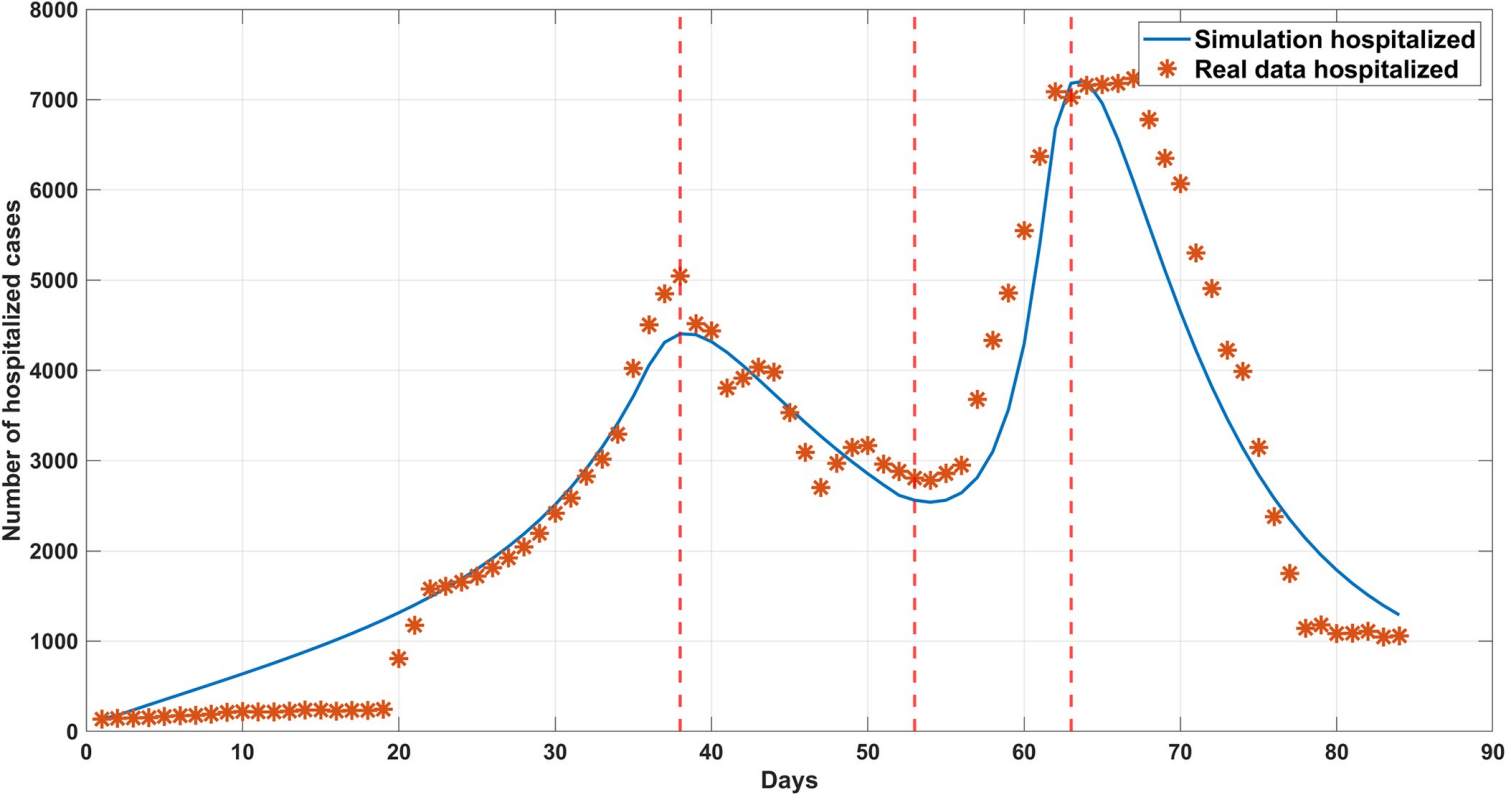
The comparison of the simulated results and the actual number of hospitalized people. The star dots represent the report daily hospitalized; blue line corresponds to the best fit of the *LUSIHR* model to the data.

The growth of the number of infected people explained by the *LUSIHR* model was calculated based on the obtained parameters discussed above. The results are shown in Fig 9. The figure shows the comparison of the simulated results to the actual number of hospitalized people.

Fig 9 shows that, in the first interval, the number of infected people increased following an exponential curve with a low power. In the third interval, the number of infected people increased exponentially with a higher power. The second interval showed a decay curve, while the actual data showed some fluctuations due to the control policy and incomplete data. Finally, the last interval exhibits the exponential decay rapidly with the lockdown policy. The simulated results of the model illustrated that the proposed model and the obtained parameters explained the spread of the COVID-19 infections in Thailand exceptionally well. Later, the model could be used to simulate future conditions and analyze different scenarios to control the virus spread resulting from undocumented immigration across the natural border.

### 4.7 Different control rates for undocumented immigration

**Fig 10:**
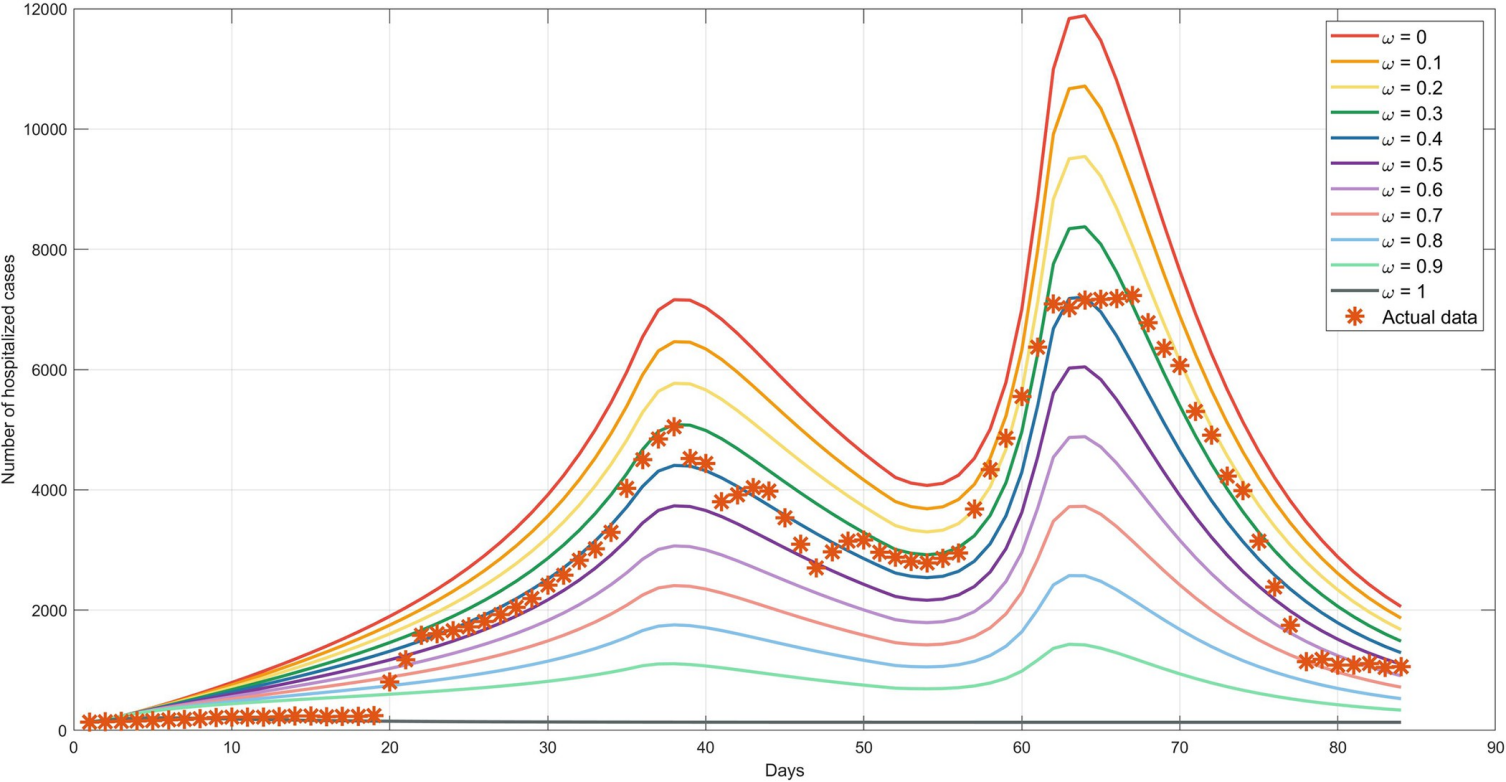
The comparison of actual data and simulation results with the different values for the control rate of undocumented immigration across the natural border (*ω*). The star dots represent the report daily hospitalized; The colored lines correspond to the different values of the control rate of undocumented immigration across the natural border (*ω*).

The spread of COVID-19 was analyzed with different control rates of undocumented immigration across the natural border to determine the effects of the epidemic. The control rate was specified by the parameter *ω*. The higher the value of *ω*, the greater the rate of arrests of undocumented immigrants crossing the natural border. For current measures, we set *ω* = 0.4, which corresponded to the actual data for the control rate of the illegal immigration in Thailand (see Fig 10). If the control rate of undocumented immigration across the natural border was less than 0.4, the virus spread increased. Fig 10 shows the results of different values for *ω* ranging from 0 to 1. The analysis of the control rates with the number of hospitalized people from the simulation results provides us a guide for decreasing the number of patients with different control rates (S1 Appendix: Appendix A5 and Table A5).

According to the resulting curves in Fig 10, fully controlled undocumented immigration (*ω* = 1) reduced the number of infected people, yielding a basic reproduction number of *R*_0_ = 0.2349 at the last interval. If we can maintain this policy over time, infected people would disappear from the system because the DFE and the epidemic could not spread. However, some levels of control could maintain conditions in a safe zone. The study indicated that when the control rate of the undocumented immigrants reached below or equal to 50% (*ω* ≤ 0.5), the number of cases in a new peak is expected to rise by at least 60% or 0.6-fold increase in the number of the patient (see the control rate analysis in **S1 Appendix**: Appendix A5 and Table A5). Although the control rate of 100% (*ω* = 1) would be the only method to reduce the number of patients in the second wave, the control rate of 90% (*ω* = 0.9) could also greatly alleviate the impact of the undocumented immigration. The maximum number of people hospitalized in a day would then decrease from about 4,406 to 1,108 cases in the first wave (Day 38) and from 7,208 to 1,431 cases in the second wave (Day 63). The analysis of the control rate (in Appendix A5 in **S1 Appendix**) also suggests that the undocumented immigrants were controlled by 80% (*ω* = 0.8), the impact of COVID-19 would be reduced and the number of new cases in the second wave would rise lower than 50% compared to that of the first wave.

## 5. Conclusion

In this study, we proposed a model for the COVID-19 epidemic in Thailand and the effects of legal and illegal immigration. This model consisted of six subpopulations: legal immigration, undocumented immigration across natural border, susceptible people, infected people, hospitalized people, and recovered people. The control rates of unlawful immigration across the natural border were evaluated under two scenarios: 0≤*ω*<1 for the *LUSIHR* model, and *ω* = 1 for the *LSIHR* model. The model parameters were adjusted to identify the best values consistent with the actual data by minimizing the RMSE. We analyzed the positivity of the model, the equilibrium points, and the basic reproduction number (*R*_0_). The analysis of equilibrium points was performed for both models. One EE point was obtained in the *LUSIHR* model in which the infection still appears. Two equilibrium points were obtained in the *LSIHR* model, the DFE and the EE. The simulations showed that the trajectory of the solutions was in the direction of the analyzed equilibrium points. For the actual data analysis, the obtained values for the parameters were used in the *LUSIHR* model, and the resulting simulation fit the real data. Finally, the effect of the different rates for controlling illegal or undocumented immigration at the natural border was compared to the actual data. This study provides a guide to the control strategies for the immigration in term of the effective control rate which can be varied. If we could not completely control the undocumented immigrants, nearly full controls more than 80%-90% could help to reduce the number of overall tendencies of the disease spread.

## 6. Discussion

The effective control rate of the undocumented immigrants was still arbitrary. However, the model could suggest that if we could control more than 80%-90%, we could reduce the number of hospitalized people and the spread could be under control. As shown in the result, an effective control rate of 90% can reduce the maximum number of hospitalized cases down for both first and second waves of the virus spread. Notably, the actual data was then used as a reference data to compare the fitting and simulation results via the proposed models. Therefore, the 90% control of the illegal immigrants here is a relative claim that can be optimized when the reference data is changed. However, the simulation results reveal that if there is a better control of the illegal immigrants to maintain the number of hospitalized people lower the reference data, it will decelerate the number of cases and only the domestic events will cause the spread of the disease.

The *LUSIHR* model provides us both theoretical analysis and real-data analysis to investigate the spread of the COVID-19 within Thailand. This proposed model is well-suited to explain the spread of COVID-19 in Thailand related to immigration across the natural border under uncontrolled, partially controlled, or fully controlled conditions (based on the control rate *ω*). In addition, the model can be applied to other communities to better understanding the spread of the virus with the time evolution considering the immigration from other countries. This model might be applied to mitigate the COVID-19 spread associated with uncontrolled international immigration as it is now rising globally [25]. Indeed, our study pointed out the significant impacts of the undocumented worker who could be COVID-19 carriers and this could bring a new wave of COVID-19 to a country in which the immigration control measures are compromised. Basically, the study suggests that when the control of undocumented immigrants reached less than 80%, the rise of COVID-19 cases related to the immigrant works would be expected. It would be necessary for the government to establish a policy in order to quickly register and enroll undocumented workers for COVID-19 screening. Obviously, the model successfully simulated peaks and the sizes of the infected population based on the given reliable data. Our results show that the *LUSIHR* and the *LSIHR* models are suitable to explain the epidemic trend due to the spread of the disease.

The *LUSIHR* model is suitable for simulating the population growth with the case of the illegal immigration in Thailand but the control rate of undocumented is hard to obtain. We obtained only the report that how many people were arrested for crossing the natural border not for the number of people who can enter the country. The hardest step for both models is to obtain the values related to the infected individuals which there is no any report. In this study, the analyzed data are taken in between December 2020 and February 2021 that are the datasets from the early detection of the first case infected immigrant into Thailand to the time of the implementation of strict control via the lockdown policy. After that, the strict lockdown policies were relieved and caused a new wave of the COVID-19 spread. Different strains of the COVID-19 are found in different area, and it causes a new wave of infection. Therefore, our model can be applied to the lockdown situation as well; especially, in the case of a widespread of the COVID-19 from the immigrants via uncontrolled natural borders. Since the different COVID-19 strains cause different infection rates [26], a family of the model can be created to foretell the situation. Thus, the modification of the models can be applied to fit with such a change of the situation. The *LUSIHR* model used here can be a base starting model to overcome the control strategies of the immigration into the country. Finally, the model can be a good hint to test various strategies to control the spread of COVID-19 not only in Thailand but also all around the world.

## Supporting information

S1 Table The data of the cumulative COVID-19 infected cases in Thailand. (XLSX)

S1 Appendix A1: Description of the data in S1 Table. A2: The proof of model positivity. A3: The analysis of the equilibrium points. A4: The local stability and the basic reproduction number. A5: The control rate (*ω*) analysis.
(PDF)
