# Supplementary material for: Modeling the spread of COVID-19 as a consequence of undocumented immigration toward the reduction of daily hospitalization: Case reports from Thailand
Source: PLoS One. 2022 Aug 25;17(8):e0273558. doi: 10.1371/journal.pone.0273558 (PMC9409513; doi:10.1371/journal.pone.0273558)
Supplement: S1 Appendix — A1: Description of the data in S1 Table. A2: The proof of model positivity. A3: The analysis of the equilibrium points. A4: The local stability and the basic reproduction number. A5: The control rate (ω) analysis. (PDF) [file pone.0273558.s002.pdf]

PLoS One Supporting Information Appendix

Article Title: Modeling the spread of COVID-19 as a consequence of undocumented immigration toward the reduction of daily hospitalization: case reports from Thailand

Authors: Tanatorn Intarapanya, Apichat Suratanee, Sittiporn Pattaradilokrat, and Kitiporn Plaimas

Appendix A1: Description of the data in S1 Table.

The data we used were described as follows:

i) Hospitalized: this is the daily hospitalized people, and we used this data for fitting parameters and simulation as an actual data.
ii) Total hospitalized: this data is the cumulative number of hospitalized people because the report from the government is the number of cases that treat in the hospital.
iii) Recovered: this is the cumulative number of recovered people, and we used this data to calculate the recovery rate of the hospitalized.
iv) Death: this is the cumulative number of deaths, and we used this data to calculate the death rate of the hospitalized.

Appendix A2: The proof of model positivity

For the *LUSIHR* model, to prove that their solutions were non-negative (all populations more than or equal to zero) if the initial conditions were non-negative, we can apply the concept of the proof the model positivity of the feedback vaccination law for SIR model [19]. The population functions $L(t)$, $U(t)$, $S(t)$, $I(t)$, $H(t)$, and $R(t)$ are continuous and differentiable as shown in Equations Error! Reference source not found. - Error! Reference source not found. in the main manuscript. Then, we need to show that if the population decreased to zero, the derivative was positive to increase the population to be positive again.

Let us consider each equation in the proposed system of equations as the following:

For the $LUSIHR$ model that $0 \le \omega < 1$,

Equation Error! Reference source not found. is for legal immigration $L(t)$ and $L'(t) = \Lambda_1 - \alpha_1 L(t) - \alpha_2 L(t)$.

Let the initial condition of the population be positive, $L(0) \ge 0$. For some t_l that $L(t_l) = 0$, then $L'(t_l) = \Lambda_1 > 0$ since Λ_1 is the recruited rate that must be greater than zero. As the derivative of L for t_l is positive, $L(t_l^+) > 0$, we have proved that $L(t) \ge 0$ for all $t \ge 0$.

Equation Error! Reference source not found. is for undocumented immigration over the natural border $U(t)$ and $U'(t) = \Lambda_2(1-\omega) - \alpha_3 U(t) - \alpha_4 U(t)$.

Let the initial condition of the population be positive, $U(0) \geq 0$. If $U(t_u) = 0$ for some $t_u > 0$, then $U'(t_u) = \Lambda_2(1-\omega) \geq 0$ since Λ_2 is the recruited rate and $(1-\omega)$ is between 0 and 1. As the derivative of U related to t_u is positive, $U(t_u^+) \geq 0$, we have proved that $U(t) \geq 0$ for all $t \geq 0$.

Equation Error! Reference source not found. is for susceptible population $S(t)$ and $S'(t) = \alpha_1 L(t) + \alpha_3 U(t) - \beta S(t) I(t) - \mu S(t)$.

Let the initial condition of the population be positive, $S(0) \geq 0$. If $S(t_s) = 0$ for some $t_s > 0$, then $S'(t_s) = \alpha_1 L(t_s) + \alpha_3 U(t_s)$. Since $L(t) \geq 0$ and $U(t) \geq 0$ for all $t \geq 0$, $\alpha_1 > 0$ and $\alpha_3 > 0$, we have $S'(t_s) \geq 0$. So $S(t_s^+) \geq 0$ and have proved that $S(t) \geq 0$ for all $t \geq 0$.

Equation Error! Reference source not found. is for infected population $I(t)$ and $I'(t) = \alpha_4 U(t) + \beta S(t) I(t) - \delta I(t) - \varepsilon_1 I(t) - \gamma_1 I(t) - \mu I(t)$.

Let the initial condition of the population be positive, $I(0) \geq 0$. If $I(t_i) = 0$ for some $t_i > 0$, then $I'(t_i) = \alpha_4 U(t_i)$. Since $U(t) \geq 0$ for all $t \geq 0$ and $\alpha_4 > 0$, we have $I'(t_i) \geq 0$. So $I(t_i^+) \geq 0$ and have proved that $I(t) \geq 0$ for all $t \geq 0$.

Equation Error! Reference source not found. is for the hospitalized population $H(t)$ and $H'(t) = \alpha_2 L(t) + \delta I(t) - \varepsilon_2 H(t) - \gamma_2 H(t)$.

Let the initial condition of the population be positive, $H(0) \geq 0$. If $H(t_h) = 0$ for some $t_h > 0$, then $H'(t_h) = \alpha_2 L(t_h) + \delta I(t_h)$. Since $L(t) \geq 0$ and $I(t) \geq 0$ for all $t \geq 0$ and $\alpha_2, \delta > 0$, we have $H'(t_h) \geq 0$. So $H(t_h^+) \geq 0$ and have proved that $H(t) \geq 0$ for all $t \geq 0$.

Equation Error! Reference source not found. is for the recovered population $R(t)$ and $R'(t) = \gamma_1 I(t) + \gamma_2 H(t) - \mu R(t)$.

Let the initial condition of the population be positive, $R(0) \geq 0$. If $R(t_r) = 0$ for some $t_r > 0$, then $R'(t_r) = \gamma_1 I(t_r) + \gamma_2 H(t_r)$. Since $I(t) \geq 0$ and $H(t) \geq 0$ for all $t \geq 0$ and $\gamma_1, \gamma_2 > 0$, we have $R'(t_r) \geq 0$. So $R(t_r^+) \geq 0$ and have proved that $R(t) \geq 0$ for all $t \geq 0$.

For the *LSIHR* model where $\omega = 1$, the positivity of $L(t)$, $H(t)$ and $R(t)$ is shown in the *LUSIHR* model. So, we will prove only $S(t)$ and $I(t)$.

Equation Error! Reference source not found. is for susceptible population $S(t)$ and $S'(t) = \alpha_1 L(t) - \beta S(t) I(t) - \mu S(t)$.

Let the initial condition of the population be positive, $S(0) \geq 0$. If $S(t_s) = 0$ for some $t_s > 0$, then $S'(t_s) = \alpha_1 L(t_s)$. Since $L(t) \geq 0$ for all $t \geq 0$, $\alpha_1 > 0$, we have $S'(t_s) \geq 0$. So $S(t_s^+) \geq 0$ and have proved that $S(t) \geq 0$ for all $t \geq 0$.

Equation Error! Reference source not found. is for the infected population $I(t)$ and $I'(t) = \beta S(t)I(t) - \delta I(t) - \varepsilon_1 I(t) - \gamma_1 I(t) - \mu I(t)$.

Let the initial condition of the population be positive, $I(0) \geq 0$. If $I(t_i) = 0$ for some $t_i > 0$, then $I'(t_i) = 0$. Therefore, $I(t_i^+) = 0$ and we proved that $I(t) \geq 0$ for all $t \geq 0$.

In conclusion, from the above analysis, all the solutions are non-negative for all times t and these proposed models can be applied to analyze its equilibrium points further and simulate and predict the growth of the populations.

Appendix A3: The analysis of an equilibrium point

For the *LUSIHR* model, the equilibrium point was calculated by setting Equations **Error! Reference source not found. - Error! Reference source not found.** equal to zero as in the following non-linear system:

$$L'(t) = \Lambda_1 - \alpha_1 L(t) - \alpha_2 L(t) = 0 \tag{1}$$

$$U'(t) = \Lambda_2 (1-\omega) - \alpha_3 U(t) - \alpha_4 U(t) = 0 \tag{2}$$

$$S'(t) = \alpha_1 L(t) + \alpha_3 U(t) - \beta S(t)I(t) - \mu S(t) = 0 \tag{3}$$

$$I'(t) = \alpha_4 U(t) + \beta S(t)I(t) - \delta I(t) - \varepsilon_1 I(t) - \gamma_1 I(t) - \mu I(t) = 0 \tag{4}$$

$$H'(t) = \alpha_2 L(t) + \delta I(t) - \varepsilon_2 H(t) - \gamma_2 H(t) = 0 \tag{5}$$

$$R'(t) = \gamma_1 I(t) + \gamma_2 H(t) - \mu R(t) = 0 \tag{6}$$

The solution of the system of Equations (1) - (6) was an equilibrium point. We obtained only one EE point in which the infection was not removed from the system; this is shown as the following.

We denote the EE point as $(L^*, U^*, S^*, I^*, H^*, R^*)$.

By Equation (1): $L'(t) = \Lambda_1 - \alpha_1 L(t) - \alpha_2 L(t) = 0$, we obtain $L^* = \dfrac{\Lambda_1}{\alpha_1 + \alpha_2}$.

By Equation (2): $U'(t) = \Lambda_2 (1-\omega) - \alpha_3 U(t) - \alpha_4 U(t) = 0$, we obtain $U^* = \dfrac{\Lambda_2 (1-\omega)}{\alpha_3 + \alpha_4}$.

By Equation (4): $I'(t) = \alpha_4 U(t) + \beta S(t)I(t) - \delta I(t) - \varepsilon_1 I(t) - \gamma_1 I(t) - \mu I(t) = 0$,
we obtain

$$(\delta + \varepsilon_1 + \gamma_1 + \mu - \beta S^*)I^* = \alpha_4 U^*$$

$$I^* = \frac{(\alpha_4 U^*)}{(\delta + \varepsilon_1 + \gamma_1 + \mu - \beta S^*)} \tag{7}$$

and denote $A = \delta + \varepsilon_1 + \gamma_1 + \mu$ where $A - \beta S^* > 0$.

By Equation (3): $S'(t) = \alpha_1 L(t) + \alpha_3 U(t) - \beta S(t) I(t) - \mu S(t) = 0$ we obtain

$$(\beta I^* + \mu) S^* = \alpha_1 L^* + \alpha_3 U^*$$

and denote $B = \alpha_1 L^* + \alpha_3 U^*$.

Substituting Equation (7) in the previous equation, we obtain

$$\frac{\alpha_4 \beta U^* S^*}{A - \beta S^*} + \mu S^* = B$$

Since $A - \beta S^* > 0$,

$$\alpha_4 \beta U^* S^* + \mu S^* (A - \beta S^*) = B(A - \beta S^*)$$

$$\mu \beta S^{*2} - (\mu A + \beta B + \alpha_4 \beta U^*) S^* + AB = 0$$

and we denote $C = \mu A + \beta B + \alpha_4 \beta U^*$.

By solving the value of S^* and $C^2 - 4\mu\beta AB > 0$, we obtain

$$S^* = \frac{C \pm \sqrt{C^2 - 4\mu\beta AB}}{2\mu\beta}$$

Since we had two values of S^*, the value of I^* from Equation (7) must have two values as follows.

$$I^* = \frac{\alpha_4 U^*}{A - \left(\dfrac{C \pm \sqrt{C^2 - 4\mu\beta AB}}{2\mu} \right)}$$

From model positivity, $I^* \geq 0$.

Notice that $A-\left(\dfrac{C\pm\sqrt{C^2-4\mu\beta AB}}{2\mu}\right)>0$ are the conditions for each equilibrium point. We obtain that the equilibrium point $I^*_1=\dfrac{\alpha_4 U^*}{A-\left(\dfrac{C+\sqrt{C^2-4\mu\beta AB}}{2\mu}\right)}$ exists if $\dfrac{2\mu A-C}{\sqrt{C^2-4\mu\beta AB}}>1$, and the equilibrium point $I^*_2=\dfrac{\alpha_4 U^*}{A-\left(\dfrac{C-\sqrt{C^2-4\mu\beta AB}}{2\mu}\right)}$ exists if $\dfrac{2\mu A-C}{\sqrt{C^2-4\mu\beta AB}}>-1$.

We will show that the condition will always be $-1<\dfrac{2\mu A-C}{\sqrt{C^2-4\mu\beta AB}}<1$.

Suppose that $\dfrac{2\mu A-C}{\sqrt{C^2-4\mu\beta AB}}\geq 1$.

We obtain $2\mu A-C\geq\sqrt{C^2-4\mu\beta AB}$. Since $C=\mu A+\beta B+\alpha_4\beta U^*$, we then get

$$2\mu A-(\mu A+\beta B+\alpha_4\beta U^*)\geq\sqrt{C^2-4\mu\beta AB}.$$

Notice that $\sqrt{C^2-4\mu\beta AB}>0$. Therefore, we take a power of two and then we get

$$\left[\mu A-\left(\beta B+\alpha_4\beta U^*\right)\right]^2\geq\left[\mu A+\left(\beta B+\alpha_4\beta U^*\right)\right]^2-4\mu\beta AB.$$

We rearrange the above expression, and we get $-4\mu A(\beta B+\alpha_4\beta U^*)\geq-4\mu\beta AB$.

Thus, $\mu A\alpha_4\beta U^*\leq 0$ and then $\dfrac{\mu(\delta+\varepsilon_1+\gamma_1+\mu)\alpha_4\beta\Lambda_2(1-\omega)}{\alpha_3+\alpha_4}\leq 0$.

Since $\mu,\alpha_3,\alpha_4,\delta,\varepsilon_1,\gamma_1,\beta,\Lambda_2>0$, and $0\leq\omega<1$, this is a contradiction. Hence, $\dfrac{2\mu A-C}{\sqrt{C^2-4\mu\beta AB}}<1$.

Suppose that $\dfrac{2\mu A-C}{\sqrt{C^2-4\mu\beta AB}}\leq -1$.

We obtain $2\mu A-C\leq-\sqrt{C^2-4\mu\beta AB}$. Since $C=\mu A+\beta B+\alpha_4\beta U^*$, we then get

$$2\mu A-(\mu A+\beta B+\alpha_4\beta U^*)\leq-\sqrt{C^2-4\mu\beta AB}.$$

Notice that $-\sqrt{C^2 - 4\mu\beta AB} < 0$. Therefore, when we take a power of two and we get

$$\left[\mu A - \left(\beta B + \alpha_4 \beta U^*\right)\right]^2 \geq \left[\mu A + \left(\beta B + \alpha_4 \beta U^*\right)\right]^2 - 4\mu\beta AB,$$

which is the same inequality as the above situation. Thus, it is a contradiction and we can conclude that $\frac{2\mu A - C}{\sqrt{C^2 - 4\mu\beta AB}} > -1$.

Therefore, we have only one equilibrium point that satisfies the system of the equation as

$$S^* = \frac{C - \sqrt{C^2 - 4\mu\beta AB}}{2\mu\beta} \text{ and } I^* = \frac{\alpha_4 U^*}{A - \beta S^*}.$$

By Equation (5): $H'(t) = \alpha_2 L(t) + \delta I(t) - \varepsilon_2 H(t) - \gamma_2 H(t) = 0$, we obtain $H^* = \frac{\alpha_2 L^* + \delta I^*}{\varepsilon_2 + \gamma_2}$.

By Equation (6): $R'(t) = \gamma_1 I(t) + \gamma_2 H(t) - \mu R(t) = 0$, we obtain $R^* = \frac{\gamma_1 I^* + \gamma_2 H^*}{\mu}$.

The EE point of the $LUSIHR$ model is

$$(L, U, S, I, H, R) = (L^*, U^*, S^*, I^*, H^*, R^*)$$

where $L^* = \frac{\Lambda_1}{\alpha_1 + \alpha_2}$, $U^* = \frac{\Lambda_2(1-\omega)}{\alpha_3 + \alpha_4}$, $S^* = \frac{C - \sqrt{C^2 - 4\mu\beta AB}}{2\mu\beta}$, $I^* = \frac{\alpha_4 U^*}{A - \beta S^*}$, $H^* = \frac{\alpha_2 L^* + \delta I^*}{\varepsilon_2 + \gamma_2}$, and $R^* = \frac{\gamma_1 I^* + \gamma_2 H^*}{\mu}$, with $A = \delta + \varepsilon_1 + \gamma_1 + \mu$, $B = \alpha_1 L^* + \alpha_3 U^*$ and $C = \mu A + \beta B + \alpha_4 \beta U^*$.

For the *LSIHR* model, the equilibrium points were calculated by using Equations (1), (5) - (6) and setting the differential Equations Error! Reference source not found. - Error! Reference source not found. as zero. Therefore, we obtained

$$S'(t) = \alpha_1 L(t) - \beta S(t) I(t) - \mu S(t) = 0 \quad (8)$$

$$\text{and } I'(t) = \beta S(t) I(t) - \delta I(t) - \varepsilon_1 I(t) - \gamma_1 I(t) - \mu I(t) = 0. \quad (9)$$

The solutions for Equations (1), (5) - (6), and (8) - (9) represented an equilibrium point. We obtained two equilibrium points, one of which was the EE point and the other a DFE point as follows:

By Equation (1): $L'(t) = \Lambda_1 - \alpha_1 L(t) - \alpha_2 L(t) = 0$, we obtain $L^* = \frac{\Lambda_1}{\alpha_1 + \alpha_2}$.

By Equation (9): $I'(t) = \beta S(t)I(t) - \delta I(t) - \varepsilon_1 I(t) - \gamma_1 I(t) - \mu I(t) = 0$ where $A = \delta + \varepsilon_1 + \gamma_1 + \mu$, we obtain $I^* = 0$ or $S^* = \dfrac{A}{\beta}$.

By Equation (8): $S'(t) = \alpha_1 L(t) - \beta S(t)I(t) - \mu S(t) = 0$, we know that $S^* = \dfrac{\alpha_1 L^*}{\beta I^* + \mu}$.

If $I_1^* = 0$, then we get $S_1^* = \dfrac{\alpha_1 L^*}{\mu}$. If $S_2^* = \dfrac{A}{\beta}$, then we obtain $I_2^* = \dfrac{\alpha_1 L^*}{A} - \dfrac{\mu}{\beta}$.

By Equation (5): $H'(t) = \alpha_2 L(t) + \delta I(t) - \varepsilon_2 H(t) - \gamma_2 H(t) = 0$, we obtain $H^* = \dfrac{\alpha_2 L^* + \delta I^*}{\varepsilon_2 + \gamma_2}$.

By Equation (6): $R'(t) = \gamma_1 I(t) + \gamma_2 H(t) - \mu R(t) = 0$, we obtain $R^* = \dfrac{\gamma_1 I^* + \gamma_2 H^*}{\mu}$.

The DFE point of the $LSIHR$ model is

$$(L, S, I, H, R) = (L_1^*, S_1^*, I_1^*, H_1^*, R_1^*)$$

where $L_1^* = \dfrac{\Lambda_1}{\alpha_1 + \alpha_2}$, $S_1^* = \dfrac{\alpha_1 L^*}{\mu}$, $I_1^* = 0$, $H_1^* = \dfrac{\alpha_2 L^* + \delta I^*}{\varepsilon_2 + \gamma_2}$, and $R_1^* = \dfrac{\gamma_1 I^* + \gamma_2 H^*}{\mu}$. Note that the DFE point of the *LSIHR* should have $I^* = 0$ in all components of the DFE.

The EE point of the $LSIHR$ model is

$$(L, S, I, H, R) = (L_2^*, S_2^*, I_2^*, H_2^*, R_2^*)$$

where $L_2^* = \dfrac{\Lambda_1}{\alpha_1 + \alpha_2}$, $S_2^* = \dfrac{A}{\beta}$, $I_2^* = \dfrac{\alpha_1 L^*}{A} - \dfrac{\mu}{\beta}$, $H_2^* = \dfrac{\alpha_2 L^* + \delta I^*}{\varepsilon_2 + \gamma_2}$, and $R_2^* = \dfrac{\gamma_1 I^* + \gamma_2 H^*}{\mu}$, with $A = \delta + \varepsilon_1 + \gamma_1 + \mu$.

Appendix A4: The local stability and the basic reproduction number

For the $LUSIHR$ model, to obtain the eigenvalue, the Jacobian was built and considered at endemic equilibrium point as follows:

$$J(S,I)=\begin{bmatrix} -\alpha_1-\alpha_2 & 0 & 0 & 0 & 0 & 0 \\ 0 & -\alpha_3-\alpha_4 & 0 & 0 & 0 & 0 \\ \alpha_1 & \alpha_3 & -\beta I-\mu & -\beta S & 0 & 0 \\ 0 & \alpha_4 & \beta I & \beta S - A & 0 & 0 \\ \alpha_2 & 0 & 0 & \delta & -\varepsilon_2-\gamma_2 & 0 \\ 0 & 0 & 0 & \gamma_1 & \gamma_2 & -\mu \end{bmatrix}$$

The eigenvalues are the solution of the equation

$$\det(J-\lambda I)=(-\alpha_1-\alpha_2-\lambda)(-\alpha_3-\alpha_4-\lambda)(-\varepsilon_2-\gamma_2-\lambda)(-\mu-\lambda)(\lambda^2+p\lambda+q)=0$$

where $p=-\beta S+A+\beta I+\mu$ and $q=A\beta I-\mu BS+\mu A$.

The eigenvalues are $\lambda_1=-\alpha_1-\alpha_2<0$, $\lambda_2=-\alpha_3-\alpha_4<0$, $\lambda_3=-\varepsilon_2-\gamma_2<0$, $\lambda_4=-\mu<0$, and the roots of $\lambda^2+p\lambda+q$.

Consider $\lambda^2+p\lambda+q=0$ at the EE of *LUSIHR* which $S^*=\dfrac{C-\sqrt{C^2-4\mu\beta AB}}{2\mu\beta}$ and $I^*=\dfrac{\alpha_4 U^*}{A-\beta S^*}$, we get

$$p=A-\beta S^*+\mu+\frac{\beta\alpha_4 U^*}{A-\beta S^*} \text{ and } q=\mu\left(A-\beta S^*\right)+\frac{A\beta\alpha_4 U^*}{A-\beta S^*}.$$

In the above expression, there is no positive real root using Descartes' rule of signs if $p>0$ and $q>0$.

To prove that $A-\beta S^*>0$, we assume that $A-\beta S^*\le 0$.

$$A\le\frac{C-\sqrt{C^2-4\mu\beta AB}}{2\mu},$$

$$2\mu A\le C-\sqrt{C^2-4\mu\beta AB},$$

$$0<\sqrt{C^2-4\mu\beta AB}\le C-2\mu A,$$

$$C^2-4\mu\beta AB\le C^2-4\mu AC+4\mu^2A^2,$$

$$\beta B\ge C-\mu A,$$

$$0\ge\alpha_4\beta U^*.$$

Since $\alpha_4, \beta, U^* > 0$, this is a contradiction.

Hence, $A - \beta S^* > 0$.

For any scenario in $LUSIHR$ model, infections will always spread in a trajectory that converges at the EE point.

For the $LSIHR$, the stability of the DFE and the EE points was determined by the condition of eigenvalues [21, 22]. To obtain the eigenvalue, the Jacobian was built and considered at each equilibrium point as follows:

$$J(S,I) = \begin{bmatrix} -\alpha_1 - \alpha_2 & 0 & 0 & 0 & 0 \\ \alpha_1 & -\beta I - \mu & -\beta S & 0 & 0 \\ 0 & \beta I & \beta S - A & 0 & 0 \\ \alpha_2 & 0 & \delta & -\varepsilon_2 - \gamma_2 & 0 \\ 0 & 0 & \gamma_1 & \gamma_2 & -\mu \end{bmatrix}$$

The eigenvalues are the solution of the equation

$$\det(J - \lambda I) = (-\alpha_1 - \alpha_2 - \lambda)(-\varepsilon_2 - \gamma_2 - \lambda)(-\mu - \lambda)(\lambda^2 + p\lambda + q) = 0$$

where $p = -\beta S + A + \beta I + \mu$ and $q = A\beta I - \mu BS + \mu A$.

The eigenvalues are $\lambda_1 = -\alpha_1 - \alpha_2 < 0$, $\lambda_2 = -\varepsilon_2 - \gamma_2 < 0$, and $\lambda_3 = -\mu < 0$, which are the roots of $\lambda^2 + p\lambda + q$.

Consider $\lambda^2 + p\lambda + q = 0$ at the DFE which $S_1^* = \frac{\alpha_1 L^*}{\mu}$ and $I_1^* = 0$, we get

$$p = -\frac{\alpha_1 \beta L_1^*}{\mu} + A + \mu \text{ and } q = -\frac{\alpha_1 \beta L_1^*}{\mu} + \mu A.$$

In the above expression, there is no positive real root using Descartes' rule of signs if $p > 0$ and $q > 0$.

Therefore, the condition that makes $p > 0$ and $q > 0$ is

$$R_0 = \frac{\alpha_1 \beta L^*}{\mu A} < 1 \text{ where } L^* = L_1^* = L_2^* \text{ and } A = \delta + \varepsilon_1 + \gamma_1 + \mu.$$

In the same way with the EE, we obtain $p = \alpha_1 \beta L^*$ and $q = \alpha_1 \beta L^* - \mu A$. Then, the condition that makes $p > 0$ and $q > 0$ is $R_0 = \frac{\alpha_1 \beta L^*}{\mu A} > 1$

R_0 is the basic reproduction number, meaning the average number of cases generated per infected individual [18]. When $R_0 > 1$, infections will spread in a trajectory that converges at the EE point. However, when $R_0 < 1$, infections will decrease over time and the number of infected people will reach the DFE point.

Appendix A5: The control rate (ω) analysis

Different control rates of the undocumented immigration and the spread of the disease were analyzed to determine the effects of the epidemic. The control rate (ω) was varied from 0 to 1 by 0.1. The higher the value of ω, the greater the rate of arrests of undocumented immigrants crossing the natural border. Note that $\omega = 0.4$ corresponded to the actual data for the control rate of the illegal immigration in Thailand. The overall simulated results were shown in Fig 10 in the main manuscript. Table A5 (below) shows the maximum number (peaks) of hospitalized people from the simulation results under the first and the second waves. The result shows that when the control rate of the undocumented immigrants reached below 50%, the number of cases in the second peak is expected to rise by at least 60% increase in the number of the patient and if the undocumented immigrants were controlled by 80%, the impact of COVID-19 would be reduced and the number of new cases in the second wave would rise lower than 50% compared to that of the first wave.

Table A5: Different values of the control rate (ω) and the number of hospitalized people at the peaks of the first and the second waves of the simulation results.

Control rate (ω)	Peak of Wave 1 (patients)	Peak of Wave 2 (patients)	% Increase
0	7,161	11,885	65.97%
0.1	6,463	10,713	65.76%
0.2	5,771	9,543	65.36%
0.3	5,085	8,375	64.70%
0.4	4,406	7,208	63.60%
0.5	3,733	6,044	61.91%
0.6	3,067	4,883	59.21%
0.7	2,407	3,725	54.76%
0.8	1,754	2,573	46.69%
0.9	1,108	1,431	29.15%
1	139	135	-2.88%
